# Expanding the Reach of Targeted Protein Degradation: The Golgi Apparatus as a New Frontier

**DOI:** 10.1002/cbic.70593

**Published:** 2026-11-14

**Authors:** Sofia Murteira, Klara Schöttgen, Marta Artola, Doris Hellerschmied

**Affiliations:** ^1^ Department of Mechanistic Cell Biology Center of Medical Biotechnology Faculty of Biology University of Duisburg‐Essen Essen Germany; ^2^ Department of Bio‐organic Synthesis Leiden Institute of Chemistry Leiden University Leiden Netherlands

**Keywords:** Golgi protein homeostasis, Golgi‐targeted protein degradation, GolgiTACs, intracellular trafficking, PROTACs

## Abstract

Targeted protein degradation (TPD) is a rapidly evolving field that has expanded the druggable proteome by enabling the selective elimination of proteins across diverse subcellular compartments. Recent mechanistic advances have shed light on protein degradation pathways operating at the Golgi apparatus, creating new opportunities to exploit these processes in chemical biology approaches and eventually for therapeutic intervention. Hijacking Golgi‐associated degradation mechanisms and adapting established TPD modalities will provide a framework to selectively target both Golgi‐resident proteins and numerous secretory and transmembrane proteins that traffic through the Golgi during biosynthesis and maturation. Here, we review recent progress in the field, discuss the unique opportunities and challenges of hijacking Golgi‐associated degradation strategies, and outline future directions for developing the next‐generation of TPD modalities.

## Graphical abstract

This perspective highlights emerging opportunities and key challenges in Golgi‐targeted protein degradation (Golgi‐TPD), informed by recent mechanistic insights into physiological Golgi degradation pathways, and provides a comprehensive resource on the Golgi proteome.

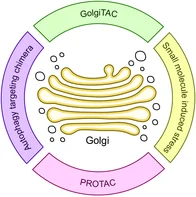

## 1 Introduction

Targeted protein degradation (TPD) has emerged as a transformative therapeutic modality that can overcome several of the limitations associated with classical occupancy‐driven inhibitors and allosteric modulators. Rather than transiently blocking protein function, TPD technologies eliminate the target protein, offering the potential for sustained pharmacological effects, improved target selectivity, overcoming target resistance, and access to previously undruggable targets.

The first examples of TPD, proteolysis‐targeting chimeras (PROTACs), pioneered more than 25 years ago by the groups of Craig Crews and Raymond Deshaies [1, 2], have now entered a new era of clinical translation, culminating in the recent FDA approval of the first PROTAC, Arvinas and Pfizer’s estrogen receptor degrader Vepdegestrant (Veppanu), for the treatment of advanced or metastatic breast cancer [3]. PROTACs are heterobifunctional molecules composed of a ligand for the protein of interest (POI) connected through a linker to a ligand that recruits an E3 ubiquitin ligase. By inducing the formation of a ternary complex between the target protein and the E3 ligase, PROTACs promote targeted protein polyubiquitination and subsequent degradation by the proteasome [1]. In parallel, molecular glues represent a complementary class of degraders that induce or stabilize protein–protein interactions between a target protein and an E3 ligase without requiring a classical bifunctional architecture [4, 5]. While proteasome‐based degradation strategies have proven highly effective for cytosolic and nuclear proteins, they are inherently limited to proteins accessible to the ubiquitin‐proteasome system (UPS). To address extracellular and plasma membrane‐associated targets, lysosome‐based degradation technologies have recently emerged. Pioneered by Carolyn Bertozzi and colleagues, lysosome‐targeting chimeras (LYTACs) expanded the scope of TPD to secreted and transmembrane proteins [6, 7, 8]. LYTACs consist of a POI ligand linked to a ligand for an endocytic trafficking receptor, thereby directing bound cargo to the lysosome for degradation. In parallel, several autophagy‐based degradation strategies, including AUTACs [9, 10], AUTOTACs [11], and ATTECs [12, 13, 14, 15], have demonstrated that intracellular proteins can also be selectively eliminated by recruiting components of the autophagy‐lysosome pathway [16, 17]. Together, these advances demonstrate that endogenous degradative pathways—including the UPS, endolysosomal trafficking, and autophagy—can all be pharmacologically harnessed to eliminate disease‐causing proteins. However, expanding TPD beyond cytosolic, nuclear, and extracellular targets continues to be challenging. In particular, the targeted degradation of organelle‐resident proteins such as those localized in the endoplasmic reticulum (ER) and Golgi apparatus remains largely unexplored despite these proteins representing a large fraction of the cellular proteome with increasing importance in physiology and disease.

The Golgi apparatus plays a central role in cellular homeostasis by orchestrating protein sorting, lipid biosynthesis, and complex post‐translational modifications [18, 19, 20]. This highly dynamic organelle receives newly synthesized proteins from the ER at the cis‐Golgi cisternae and traffics them along the medial‐ and trans‐Golgi cisternae before reaching the trans‐Golgi network (TGN). From the TGN, proteins are sorted to their final destinations, including endosomes, lysosomes, the plasma membrane, or the extracellular space. The Golgi harbors a remarkably diverse proteome, comprising both Golgi‐resident proteins and cargo proteins in transit. To provide a systematic overview of this protein landscape, we compiled publicly available experimental datasets with subcellular resolution to establish a scored Golgi proteome (see Table S1 for datasets and scoring criteria). Golgi‐resident proteins include large families of glycosyltransferases and glycosidases that sequentially assemble, trim, and remodel N‐ and O‐linked glycans, glycolipids, and proteoglycans as cargo traverses the Golgi apparatus [21, 22, 23]. Additional biosynthetic components, including sulfotransferases [24, 25], lipid‐modifying enzymes [26], nucleotide‐sugar transporters (e.g., members of the SLC35 family) and ion transporters that regulate Golgi homeostasis (e.g., TMEM165 and SPCA1) [27] establish the biochemical environment required for glycan and lipid maturation. Beyond this biosynthetic machinery, the Golgi apparatus contains an extensive network of structural and regulatory proteins that maintain organelle architecture and coordinate vesicular trafficking. These include golgins, tethering complexes [28], and soluble NSF attachment protein receptors (SNAREs) [29], coat proteins, and regulatory small GTPases of the ARF and Rab family, which collectively control membrane identity, vesicle formation, cargo sorting, and Golgi dynamics [30, 31, 32, 33]. Finally, cargo receptors and sorting factors ensure the efficient transport of proteins and lipids throughout the secretory pathway and to their appropriate cellular destinations [19]. Despite continuous membrane flux and cargo transport, Golgi‐resident proteins are maintained within their respective cisternae through a combination of retention, retrieval, and recycling mechanisms [34]. These dynamic processes preserve the Golgi identity and enable the compartmentalized enzymatic reactions that regulate protein maturation. Notably, virtually all secreted proteins and most transmembrane proteins transit through the Golgi en route to their final cellular destination, placing this organelle at the center of cellular communication, membrane homeostasis, and protein maturation.

Given its central role in protein maturation, Golgi dysfunction has been implicated in a wide range of diseases [35]. Aberrant glycosylation resulting from the altered expression, localization, or activity of Golgi‐resident enzymes is a hallmark of many cancers and contributes to tumor progression, metastasis, and immune evasion [36, 37, 38, 39]. Likewise, defects in Golgi‐associated pathways have been linked to lysosomal storage disorders, neurodegenerative diseases, viral infection, and immune dysregulation [40, 41, 42]. This highlights the therapeutic potential of selectively modulating Golgi‐resident proteins.

Importantly, the emergence of TPD has shifted the paradigm from merely inhibiting protein function to controlling protein abundance and lifetime. Unlike cytosolic proteins, Golgi proteins continuously traffic between interconnected membrane compartments, expose domains to either the cytosol or the Golgi lumen, and undergo dynamic cycles of retention, retrieval, and recycling (Figure 1). Consequently, membrane topology, trafficking itinerary, and subcellular accessibility become critical design parameters for degradation strategies. Throughout this perspective, we discuss recent advances in our understanding of physiological Golgi‐associated degradation pathways and how these unique features of Golgi biology could be exploited to develop next‐generation targeted degradation strategies for Golgi‐resident proteins and proteins in transit through the Golgi apparatus. We highlight how endogenous Golgi homeostasis mechanisms—including intracellular trafficking decisions, autophagy, and Golgi protein quality control—may be harnessed for Golgi remodeling. Finally, we discuss lysosome‐directed strategies that reroute luminal and secretory proteins through endogenous Golgi–endosome–lysosome trafficking pathways and introduce Golgi‐targeted chimeras (GolgiTACs) as a potential future degradation modality.

**FIGURE 1:**
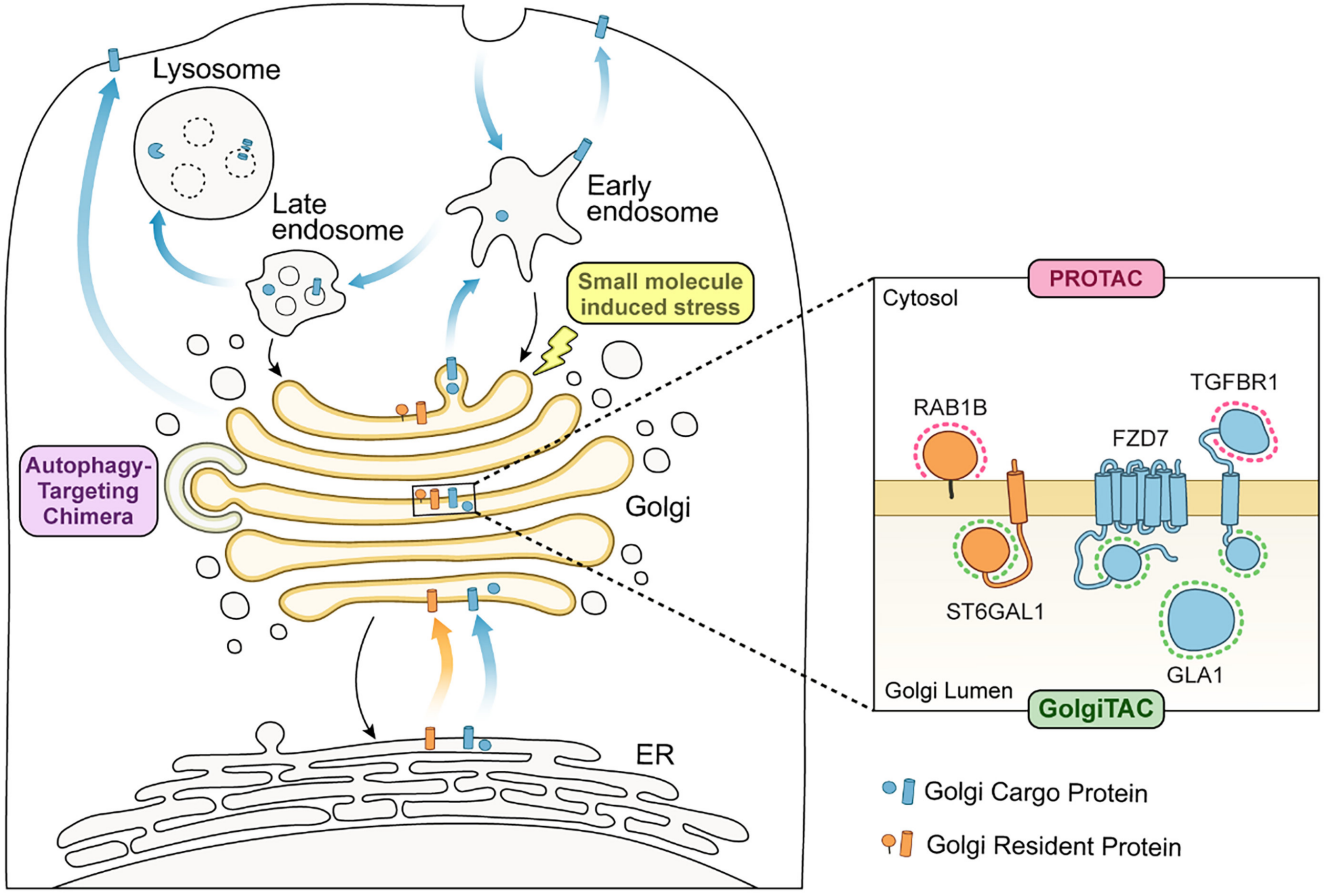
The Golgi apparatus as a landscape for targeted protein degradation. Proteins reaching the Golgi through the secretory pathway can either remain as Golgi residents or transit as cargo en route to the plasma membrane, endosomes, lysosomes, or the extracellular space. Retrograde trafficking continuously exchanges proteins between the Golgi, endosomes, and the endoplasmic reticulum. Target topology and subcellular accessibility determine which degradation technologies can be exploited. Cytosol‐exposed domains (for example, in the TGFBR1 receptor, trafficking through the Golgi, or the Golgi peripheral membrane protein and small GTPase RAB1B) may be amenable to PROTAC‐mediated proteasomal degradation, whereas autophagy‐targeting chimeras can promote lysosomal clearance of Golgi proteins or membranes, and small‐molecule‐induced Golgi stress can trigger endogenous quality‐control and degradation pathways. Luminal and secretory proteins (for example, GLA1) or transmembrane proteins with large luminal domains (for example, glycosylation enzymes as ST6GAL1 or FZD receptors on their way to the plasma membrane) may instead require rerouting through endogenous Golgi‐endosome‐lysosome trafficking pathways. This provides the conceptual basis for the future development of Golgi‐targeted chimeras (GolgiTACs), a new proposed degradation strategy for selectively targeting Golgi‐resident and secretory proteins transiting through the Golgi.

## 2 Small Molecules Inducing Golgi Protein Depletion

The Golgi apparatus is highly sensitive to cellular stress, responding with Golgi fragmentation and the redistribution of Golgi‐resident proteins to other organelles. These phenotypes have been reported under pathophysiological conditions [43, 44], and they can also be induced experimentally using small‐molecule Golgi stressors. These compounds perturb characteristic Golgi features, such as luminal pH or membrane lipid composition, thereby disrupting Golgi homeostasis. Notably, their use has linked Golgi stress to Golgi structural remodeling and the depletion of a distinct set of Golgi‐resident proteins.

In this context, early studies by Linstedt and colleagues provided important mechanistic insight into the regulation of GOLIM4 trafficking and degradation. GOLIM4, also known as GPP130, is a single‐pass transmembrane protein with a large luminal domain that shuttles between the Golgi and endosomes, with a steady‐state Golgi localization [45]. Targeting GOLIM4 has attracted considerable attention because it serves as an intracellular trafficking receptor for the bacterial Shiga toxin, mediating its endosome‐to‐Golgi transport, thereby promoting intoxication [46]. In response to manganese, GOLIM4 oligomerizes, exits the Golgi, and is targeted for lysosomal degradation, thus reducing Shiga toxin cytotoxicity [47, 48]. In addition, in response to the Golgi stressor monensin, GOLIM4 redistributes to endosomes, and its levels rapidly decrease [49, 50, 51, 52]. Broadly studying the cellular effects of Golgi stressors, including ionophores and oxysterol‐binding protein (OSBP)‐targeting compounds, has extended these phenotypes to additional Golgi‐resident proteins and established Golgi protein depletion as a common response to Golgi stress [52, 53, 54].

Ionophores (e.g., nigericin, monensin, and salinomycin) increase the pH of the Golgi lumen, resulting in swelling of the Golgi cisternae, Golgi fragmentation, and impaired Golgi trafficking [55, 56, 57, 58]. Specifically, ionophores impair the trafficking of secretory and transmembrane proteins, leading to their accumulation at the Golgi apparatus [52, 59]. Ionophores also impair endosome‐to‐Golgi trafficking, contributing to the redistribution of Golgi proteins, including GOLIM4, across the endo‐lysosomal system and in some cases promoting their depletion [56]. Additionally, the increase in luminal pH affects calcium‐binding Golgi trafficking chaperones, NUCB1 and SDF4, which exit the Golgi apparatus and are rapidly released from cells [52]. The ionophore‐induced disruption of Golgi trafficking may serve as a protective stress response mechanism that restricts the delivery of immature or damaged proteins to the plasma membrane during Golgi stress.

A second class of Golgi stressors comprises OSBP inhibitors, which disrupt lipid homeostasis. OSBP regulates the exchange of cholesterol and phosphatidylinositol 4‐phosphate (PI(4)P) between the ER and the TGN [60]. This lipid exchange maintains an optimal PI(4)P gradient along the secretory pathway, with high levels at the Golgi, which are critical for controlled protein glycosylation, vesicle formation, and protein sorting at the TGN [61]. ORPhilins are a group of natural products identified as high‐affinity ligands of OSBP and its paralog OSBP2 [62]. The ORPhilin OSW‐1 induces the localization of OSBP to TGN‐ER contact sites while inhibiting its PI(4)P‐cholesterol exchange activity and eventually inducing OSBP degradation [60, 62]. Phenotypically, OSW‐1 leads to Golgi fragmentation and impairs protein trafficking through the secretory pathway [62, 63]. More recently, two additional OSBP‐targeting compounds, oxybipins and orpinolide, were shown to induce similar Golgi stress phenotypes [53, 54]. While orpinolide and oxybipins do not induce the degradation of their direct target, OSBP, both induce the depletion of Golgi‐resident proteins. In particular, they reduce the levels of several Golgi transmembrane proteins, including GOLIM4, and other trafficking factors, as well as glycosylation enzymes [53, 54]. A similar set of Golgi proteins was depleted in response to the ionophore nigericin, suggesting that the loss of these proteins represents a shared cellular response to distinct forms of Golgi stress, rather than a compound‐specific effect [52].

Although small‐molecule Golgi stressors exhibit pleiotropic cellular effects, they have served as valuable tools in the protein degradation field. They have supported the identification of molecular mechanisms underlying the Golgi protein degradation pathways, providing the fundamental basis for their targeted modulation, as discussed in the following sections. Moreover, we propose that Golgi stressors could also aid in the development of Golgi‐TPD strategies. By inducing Golgi fragmentation, they may support the generation of Golgi fragments that are more amenable to targeted degradation approaches. Additionally, some Golgi stressors arrest proteins that are destined for the plasma membrane at the Golgi, thereby increasing their Golgi residence time and shaping the pool of potential substrates available for degradation [64]. Combining Golgi TPD strategies with carefully controlled Golgi stress may therefore provide a promising experimental avenue for defining the cellular conditions that favor Golgi membrane protein degradation.

## 3 Golgiphagy (and GOMED)

Golgi turnover and remodeling are controlled by different pathways, with the bulk degradation of Golgi membranes mediated by macroautophagy [65]. Macroautophagy (hereafter referred to as autophagy) is a process that mediates the degradation of large cytoplasmic structures, ranging from protein aggregates to damaged cellular organelles and even intracellular pathogens [66], which are generally inaccessible to the UPS. Consequently, hijacking autophagy pathways holds potential to selectively clear the cell of damaged Golgi membranes and to drive Golgi remodeling in a more controlled fashion than by the use of small‐molecule stressors.

Mechanistically, the degradation by autophagy involves the formation of a double‐membrane structure, the phagophore, which engulfs the cargo and closes around it to form the autophagosome. The closed autophagosome eventually fuses with a lysosome, where its contents are degraded [67]. Cargo selectivity is typically provided by receptors, which simultaneously engage the cargo and Atg8‐family LC3/GABARAP proteins conjugated to the growing phagophore membrane through a Ub‐like conjugation cascade [66, 68]. Phagophore biogenesis and expansion are regulated by core autophagy complexes. To this end, the activated ULK1 kinase forms a condensate at the ER and thereby serves as a scaffold for the PI3 kinase (PI3K) complex, ATG9A vesicles, the ATG2‐WIPI complex, and the LC3/GABARAP‐conjugation machinery—all of which are required for autophagosome biogenesis [68, 69]. Importantly, several autophagy receptors not only select cargo but also initiate autophagosome biogenesis by recruiting the core autophagy machinery, including the ULK1 complex through FIP200 or WIPI proteins [69].

A major recent advance in the field has been the identification of dedicated Golgiphagy receptors that mediate the selective turnover of Golgi membranes (Figure 2A). YIPF3/4 and TM9SF3 serve as integral membrane Golgiphagy receptors (Figure 2B) [70, 71, 72]. Specifically, YIPF3 and TM9SF3 contain functional LC3‐interacting region (LIR) motifs in their cytosolic tails, allowing them to engage LC3 proteins on the expanding phagophore [70, 71]. Both of these receptor‐mediated Golgiphagy pathways are triggered by nutrient stress [70, 71, 72], with Golgi and ER proteins even serving as preferred substrates of autophagic degradation during starvation [70]. TM9SF3‐dependent Golgiphagy is also induced by small‐molecule Golgi stressors such as the ionophore monensin, the trafficking inhibitor brefeldin A, α‐glucosidase inhibitors castanospermine and 1‐deoxynojirimycin, as well as the knockdown of the CMP‐sialic acid synthase CMAS or the sialic acid transporter SLC35A1 [72]. Following these exciting recent discoveries, elucidating how these integral membrane Golgiphagy receptors are activated will be critical for the future development of targeted Golgiphagy strategies.

**FIGURE 2:**
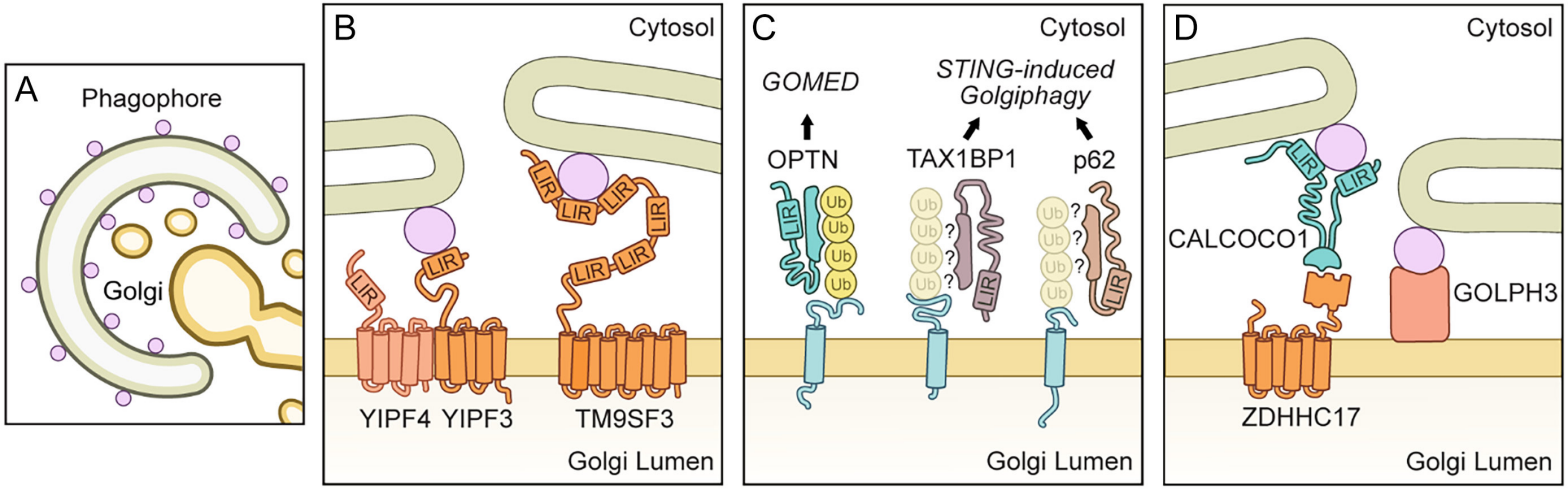
Molecular mechanisms of selective Golgiphagy. (A) The phagophore decorated with LC3‐ or GABARAP‐proteins (represented as filled purple circles) engulfs the Golgi apparatus for degradation by autophagy. Selective autophagy is facilitated by cargo receptors. (B) Integral Golgi membrane cargo receptors engage LC3‐ or GABARAP‐proteins with their LIR motifs, thereby bridging the growing phagophore to Golgi membranes. (C) Ubiquitin‐binding cargo receptors bind to ubiquitinated proteins at the Golgi. Optineurin thereby targets Golgi cargo for GOMED. TAX1BP1 and p62 are critical for Golgi degradation upon induction of the cGAS‐STING pathway. (D) Golgi resident proteins ZDHHC17 and GOLPH3 serve as assembly platforms for the autophagy machinery, including the cargo receptor CALCOCO1.

In addition to integral membrane Golgiphagy receptors, soluble cargo receptors induce the degradation of Golgi membranes. From the group of established ubiquitin‐binding cargo receptors, optineurin (OPTN), p62, and TAX1BP1 have been reported to operate at the Golgi (Figure 2C). Optineurin binds to poly‐ubiquitinated proteins—also at the Golgi—and targets them for Golgi membrane‐associated degradation (GOMED), a process related to Golgiphagy [73]. GOMED provides a means to engulf secretory and transmembrane cargo that is trapped at the Golgi [74]. During GOMED, double membrane structures originating from the TGN engulf cargo and generate autophagosome‐like structures [74]. The formation of these structures requires ULK1 and BECN1, which is part of the PI3K complex but is independent of the LC3‐conjugation machinery ATG5/7 [74].

The TAX1BP1 and p62 cargo receptors target Golgi membranes for degradation in the context of the cGAS‐STING pathway, a critical cytosolic DNA sensing pathway that induces a pro‐inflammatory response [75]. The activation of STING involves its translocation from the ER to the Golgi, where it oligomerizes and triggers a signaling cascade culminating in the activation of an inflammatory response [76]. At the same time, STING activation also serves as a Golgi stressor, inducing the formation of Golgi fragments. In turn, one mechanism that contributes to terminating STING signaling and prevents the prolonged activation of inflammatory responses is the degradation of STING and STING‐induced Golgi fragments by Golgiphagy [75]. In the context of this pathway, an important question is whether STING‐positive Golgi fragments are preferentially degraded. This would suggest that a subset of Golgi fragments can be selectively eliminated. This specific Golgiphagy pathway requires STING, TAX1BP1, and p62 but is independent of FIP200 and ATG3 (an enzyme involved in LC3 conjugation to the phagophore) and TSG101 (a key protein in the microautophagy pathway) [75]. From this molecular machinery, p62 has served as an access point to generate compounds, hijacking the autophagy machinery. AUTOphagy‐TArgeting Chimeras (AUTOTACs) are heterobifunctional molecules that bridge the p62 receptor and a POI to induce the selective autophagic degradation of the latter [11]. Their p62‐binding moiety, a chemical mimetic of an N‐terminal degron, not only recruits p62 but also activates it to facilitate autophagosome biogenesis [11]. AuTophagy‐TEthering Compounds (ATTECs), in contrast, induce cargo tethering to LC3 with the aim of recruiting maturing phagophores [11]. These modalities have afforded the autophagic degradation of oncoproteins and pathogenic intracellular protein aggregates, as well as mitochondria in the case of mito‐ATTECs [77]. However, simply tethering LC3 or the LC3‐interacting autophagy receptor p62 to Golgi membranes using induced protein dimerization was insufficient to induce Golgiphagy [78]. In addition, the AUtophagy‐TArgeting Chimera (AUTAC) strategy has also enabled targeted degradation of mitochondria [10]. Mito‐AUTACs consist of a ligand engaging a mitochondrial outer membrane protein linked to a *p*‐fluorobenzylguanine (FBnG) degradation tag [10]. The AUTAC itself induced the accumulation of poly‐ubiquitin chains and LC3 at mitochondria. Notably, AUTAC‐induced mitophagy additionally required prior mitochondrial fragmentation, providing pieces that are amenable to engulfment by autophagosomes [10]. By analogy, we propose that Golgiphagy‐inducing compounds may prove most effective in combination with Golgi stressors, which induce the formation of digestible Golgi fragments, or in disease contexts characterized by a disrupted Golgi morphology. Moreover, selectivity in hijacking Golgiphagy pathways may be achieved by distinguishing different types of Golgi fragments generated under stress or pathological conditions. To this end, identifying molecular markers of damaged Golgi membranes will be essential for the rational design of AUTOTACs directed toward the Golgi. In the design of LC3‐tethering strategies, an additional consideration is the recently described process of conjugation of LC3 to single membranes (CASM). In the case of Golgi membranes, this triggers a transcriptional Golgi stress response [79]. Once the Golgi membranes are coated with LC3, the transcription factor TFE3 translocates to the nucleus, where it specifically upregulates the expression of genes involved in Golgi vesicle transport (e.g., WIPI49 and STX3A) and Golgi structural maintenance (e.g., GCP60 and SIAT4A) to restore Golgi homeostasis [79]. This response may impact the Golgi stress state of the cell and interact with induced Golgiphagy strategies relying on LC3‐recruitment.

Finally, recent studies suggest that direct interactions between Golgi‐resident proteins and cargo receptors or the autophagy machinery may serve as additional entry points for targeted Golgiphagy strategies (Figure 2D). Under nutrient starvation, the Golgi‐resident palmitoyltransferase ZDHHC17 recruits the autophagy receptor CALCOCO1, initially identified as an ER‐phagy receptor, to induce Golgi degradation [80, 81]. Additionally, GOLPH3, a peripheral Golgi membrane protein, is involved in the recruitment of LC3 to Golgi membranes upon different types of Golgi stress, including nutrient starvation, treatment with brefeldin A, nocodazole, monensin, or nigericin [82]. Although the exact molecular mechanism remains to be determined, GOLPH3 may serve as a platform for assembling the autophagic machinery at the Golgi apparatus under stress [82]. Conceptually similar, in *Drosophila*, Golgi‐localized GMAP regulates Golgi size and morphology by Golgiphagy through a direct interaction with the fly Atg8 protein [83]. These studies highlight the direct interaction between the Atg8 family, LC3/GABARAP proteins, and Golgi‐resident proteins as a regulatory mechanism of Golgiphagy.

An in‐depth mechanistic understanding of Golgiphagy initiation and of the mode of action of Golgiphagy receptors will be key for the future development of targeted Golgiphagy strategies. Conceptually, it has become apparent that these strategies will need to satisfy two important requirements. First, the core autophagy machinery must be recruited to the Golgi apparatus and locally activated. Second, and concomitantly, the Golgi apparatus needs to be selectively engaged and delivered as digestible cargo. Developing agents fulfilling both requirements may enable the targeted degradation at the organelle level. We envision that eventually, targeted Golgiphagy strategies may be finetuned to selectively target damaged Golgi membranes or distinct classes of Golgi fragments while preserving functional Golgi compartments.

## 4 Proteasomal Degradation From the Golgi Apparatus

The proteasome has emerged as an important regulator of Golgi homeostasis [84, 85], contributing to protein quality control, organelle organization, and stress adaptation. Work by Merbl and colleagues showed that the stress‐induced proteasomal degradation of Golgi matrix proteins induces Golgi fragmentation [86]. Specifically, the peripheral Golgi membrane protein GM130, which is a key component of the Golgi matrix, undergoes proteasomal degradation in response to treatment with the sialyltransferase inhibitor lithocholylglycine, the ionophore monensin, or knockdown of sialylation factors SLC35A1 or CMAS, resulting in Golgi fragmentation, which can be counteracted by inhibiting the proteasome [86]. In addition, in the context of protein quality control, the proteasome also contributes to clearing the Golgi of damaged or orphaned proteins [87, 88], which is critical to prevent Golgi fragmentation [87].

Mechanistic insight into the ubiquitination and selective proteasomal degradation of Golgi‐localized proteins first emerged from studies in yeast, where a dedicated endosome and Golgi‐associated degradation (EGAD) machinery induces substrate ubiquitination, membrane extraction, and proteasomal degradation [85, 89]. The EGAD machinery includes the rhomboid pseudoprotease Dsc2, which is responsible for substrate recognition, and a multipass transmembrane RING‐type E3 ligase (Tul1), which catalyzes substrate ubiquitination [88, 90]. The EGAD subunit Ubx3 facilitates the recruitment of the AAA‐unfoldase Cdc48 (p97/VCP in mammals), which extracts ubiquitinated membrane proteins from the Golgi membrane for proteasomal degradation [88]. Alternatively, ubiquitinated Golgi proteins can be targeted for lysosomal degradation by the endosomal sorting complex required for transport (ESCRT) machinery [91]. Through TPD strategies in yeast, Piper and colleagues demonstrated that the fate of ubiquitinated Golgi proteins is determined by the nature of the attached ubiquitin chain [92]. Ubiquitination of a Golgi‐localized substrate containing a large luminal and cytosolic domain was induced by recruiting different soluble cytosolic E3 ligases by means of a protein dimerization approach and by the auxin‐induced degradation (AID) system [92]. While substrates modified with Lys63‐linked ubiquitin chains were recognized by the ESCRT machinery and targeted for lysosomal degradation, Lys48‐linked ubiquitin chains were engaged by ubiquitin‐dependent proteases that shear domains from the membrane in a process called the Cleavage of Ubiquitinated Targets by Ubiquitin‐dependent Proteases (CUT‐UP) pathway. The cytosolic substrate domain was subsequently degraded by the proteasome, while the luminal domain was degraded in the vacuole (the equivalent of the mammalian lysosome). This study highlights the importance of carefully choosing E3s and E2/E3 pairs in Golgi‐TPD approaches as these determine the Ub linkage type and thereby the fate of ubiquitinated Golgi proteins.

In mammalian cells, only a limited number of ubiquitin E3 ligases have been reported to operate at the Golgi apparatus [93]. However, as demonstrated in yeast and also in mammalian cells, soluble cytosolic E3 ligases—and, by extension, the upstream E1 and E2 ubiquitination machinery—can be hijacked for TPD of Golgi‐localized proteins. Given the localization of the ubiquitination machinery in the cytosol, for Golgi proteins, a ligandable cytosolic or transmembrane domain is strictly required to enable substrate engagement in a “classical” PROTAC approach (Figure 3). To date, PROTACs have successfully been applied to endogenously tagged Golgi‐fusion proteins, thereby bypassing the need for ligands binding directly to Golgi proteins. Genetic fusions with Halo‐, SNAP‐, FKBP‐, and AID‐tags afforded induced proteasomal degradation with tag‐targeting PROTACs and molecular glues [64, 87, 94, 95, 96, 97]. These studies provide important precedent, demonstrating that Golgi‐localized targets are amenable to induced proteasome‐mediated degradation. The investigated targets span from peripheral Golgi proteins to integral membrane proteins.

**FIGURE 3:**
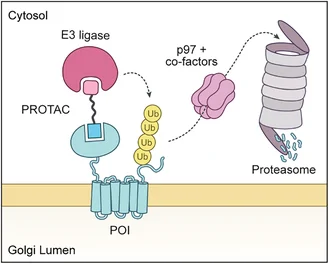
PROTAC‐induced degradation from the Golgi apparatus. PROTACs engage substrates at the cytosolic face of the Golgi and recruit ubiquitin E3 ligases. Upon formation of a ternary complex, substrate proteins are poly‐ubiquitinated and degraded by the proteasome. The proteasomal degradation of transmembrane proteins depends on their extraction by the p97 machinery.

The AID system enabled the efficient depletion of a range of peripheral Golgi proteins. With this system, an AID‐fusion protein is ubiquitinated following auxin‐induced recruitment of the CRL^TIR1^ ubiquitin ligase [98, 99]. For example, the auxin‐induced depletion of an AID‐RAB1B fusion protein led to a severe disruption of the Golgi structure and strongly impaired ER‐to‐Golgi vesicle trafficking [95]. RAB1B belongs to the family of small RAB GTPases, which are critical for Golgi‐related trafficking processes. RAB GTPases are anchored at the cytosolic side of the Golgi through C‐terminal prenylation, demonstrating that lipid‐anchored peripheral membrane proteins are amenable to TPD. Also using the AID system, the Golgi matrix proteins GRASP55 and GRASP65 were individually depleted and codepleted, respectively [97]. GRASP proteins are anchored at the Golgi membrane through N‐terminal myristoylation and are integrated via oligomerization and interactions with Golgi tethers into a protein‐rich matrix at the cytosolic face of the Golgi [100]. Acute degradation of GRASP55 and GRASP65 compromised the integrity of the Golgi ribbon, while stacking of cisternae was unaffected, demonstrating that TPD can regulate Golgi architecture [97]. Finally, the degradation of a subunit of the conserved oligomeric Golgi (COG) complex, COG4, using the AID system, led to remodeling of the Golgi, with Golgi enzymes and tethers redistributed to COG‐dependent vesicles [96]. Collectively, these studies demonstrate that the Golgi structure and trafficking can be modulated with TPD.

Multipass transmembrane proteins, specifically solute carrier (SLC) transporters, have also been degraded from the Golgi apparatus using the FKBP‐dTAG system [64]. Targets included the Golgi‐resident transporter SLC35B2 as well as SLC38A2, a transporter en route to the plasma membrane, which was accumulated at the Golgi apparatus by treatment with the ionophore monensin [64]. Using dTAGs, the SLC‐transporter‐FKBP fusion proteins were ubiquitinated by the CRL2^CRBN^ ligase and degraded by the proteasome [64]. Moreover, tagging a Golgi multipass transmembrane protein (TMEM115) with a HaloTag domain provided insight into the proteasomal degradation from the Golgi by means of hydrophobic tagging [87]. Here, a hydrophobic tag induced the misfolding of the HaloTag domain and simultaneously the exposure of a C‐terminal –GG degron sequence. This degron was recognized by the CRL substrate receptor KLHDC2 at the Golgi apparatus, resulting in substrate ubiquitination and proteasomal degradation. This study demonstrated that the exposure of a C‐degron is an efficient signal for clearing proteins from the Golgi and aligns with previous work suggesting that terminal degrons serve as degradation signals for mis‐localized or mis‐inserted proteins of the secretory pathway [101]. Once ubiquitinated, the proteasomal degradation of Golgi proteins is facilitated by the p97 machinery [86, 87], indicating a similar mechanism as observed for the PROTAC‐induced degradation from the ER [102]. We presume that at the Golgi apparatus, the PROTAC‐induced degradation would also follow a pseudocatalytic mechanism.

The successful application of the dTAG and AID systems at the Golgi demonstrates that PROTACs recruiting Cullin‐RING ligases are an effective strategy for induced Golgi‐TPD. In addition to the widely engaged CRL substrate receptors CRBN, VHL, and TIR1, Golgi‐localized substrate receptors with potential for proximity‐induced degradation include CDCA3, FBXL8, FBXW4, KLHL20, LGALS3BP, and RCBTB2. However, the substrate spectrum of soluble E3 ligases, as currently engaged by classical PROTACs, is constrained to peripheral Golgi membrane proteins and transmembrane proteins with ligandable cytosolic domains. We anticipate that the identification and characterization of integral Golgi membrane E3 ligases may further expand the substrate spectrum of Golgi‐TPD strategies. Based on their topology and subcellular localization, for example, the protease‐associated (PA)‐transmembrane (TM)‐RING ligase family holds potential for the development of future TPD strategies at the Golgi and the endo‐lysosomal system [103]. The PA‐TM‐RING family comprises 11 members, of which four (RNF13, RNF128, RNF133, and RNF150) were shown to have potential for application as proximity‐dependent degraders [104]. Notably, RNF128 and RNF133 are part of the Golgi proteome (Table S1). Conceptually similar to AbTACs, which engage the PA‐TM‐RING family member RNF43 at the plasma membrane [105], one can imagine E3‐substrate binding to occur within the Golgi lumen, while ubiquitination takes place on the cytosolic face of the Golgi apparatus. This could provide access to transmembrane proteins without ligandable cytosolic domains, including many Golgi‐localized carbohydrate‐processing enzymes, provided that suitable lysine residues are present in their cytosolic tails.

Following induced ubiquitination, the degradation pathway engaged will depend on the ubiquitin signal and the topology of the substrate protein. The extraction and proteasomal degradation of ubiquitinated Golgi multipass transmembrane proteins are feasible [64, 87], whereas substrates with large luminal domains may need to engage alternative degradation pathways. These could include targeting the ubiquitinated proteins from the Golgi to the ER, where protein channels that support retro‐translocation to the cytosol for proteasomal degradation are available [106]. Another option may be targeting to lysosomes, possibly in an ESCRT‐dependent manner, which preferentially recognizes Lys63‐linked ubiquitin chains [107]. Degradation may also involve processing by ubiquitin‐directed and intramembrane proteases, similar to the CUT‐UP pathway reported in yeast [92], which favors Lys48‐linked ubiquitin chains. In mammalian cells, the intramembrane aspartyl protease SPPL3 cleaves numerous Golgi type II transmembrane proteins, particularly glycosyltransferases, glycosidases, and sulfotransferases, within their transmembrane domains, releasing their luminal catalytic ectodomains for secretion and degradation [108, 109]. Therefore, selective proteolytic cleavage provides an additional mechanism for remodeling the Golgi proteome and regulating Golgi protein abundance.

## 5 Receptor‐Mediated Lysosomal Targeting: Golgi‐to‐Lysosome Transport

A complex network of trafficking receptors coordinates protein transport between the TGN, endosomes, and lysosomes, thereby maintaining lysosomal function and cellular proteostasis [19]. LYTACs exploit these endogenous sorting pathways to redirect extracellular and plasma membrane proteins toward lysosomal degradation [110, 111, 112]. Lysosomal targeting receptors (LTRs) currently exploited for lysosomal degradation include the cation‐independent mannose‐6‐phosphate receptor (CI‐M6PR) [6], the asialoglycoprotein receptor (ASGPR) [7], sortilin [113, 114], the transferrin receptor (TfR) [115], GLUT1 [116, 117], folate receptors (FR) [118, 119], GLP‐1R [120], cytokine receptors [121], scavenger receptors [122], and integrins [123] (Figure 4, gray pathway). Collectively, LYTACs demonstrate that the endogenous trafficking machinery can be therapeutically exploited to deliver proteins, antibodies, oligonucleotides, and other macromolecular cargos from the extracellular domain to lysosomes.

**FIGURE 4:**
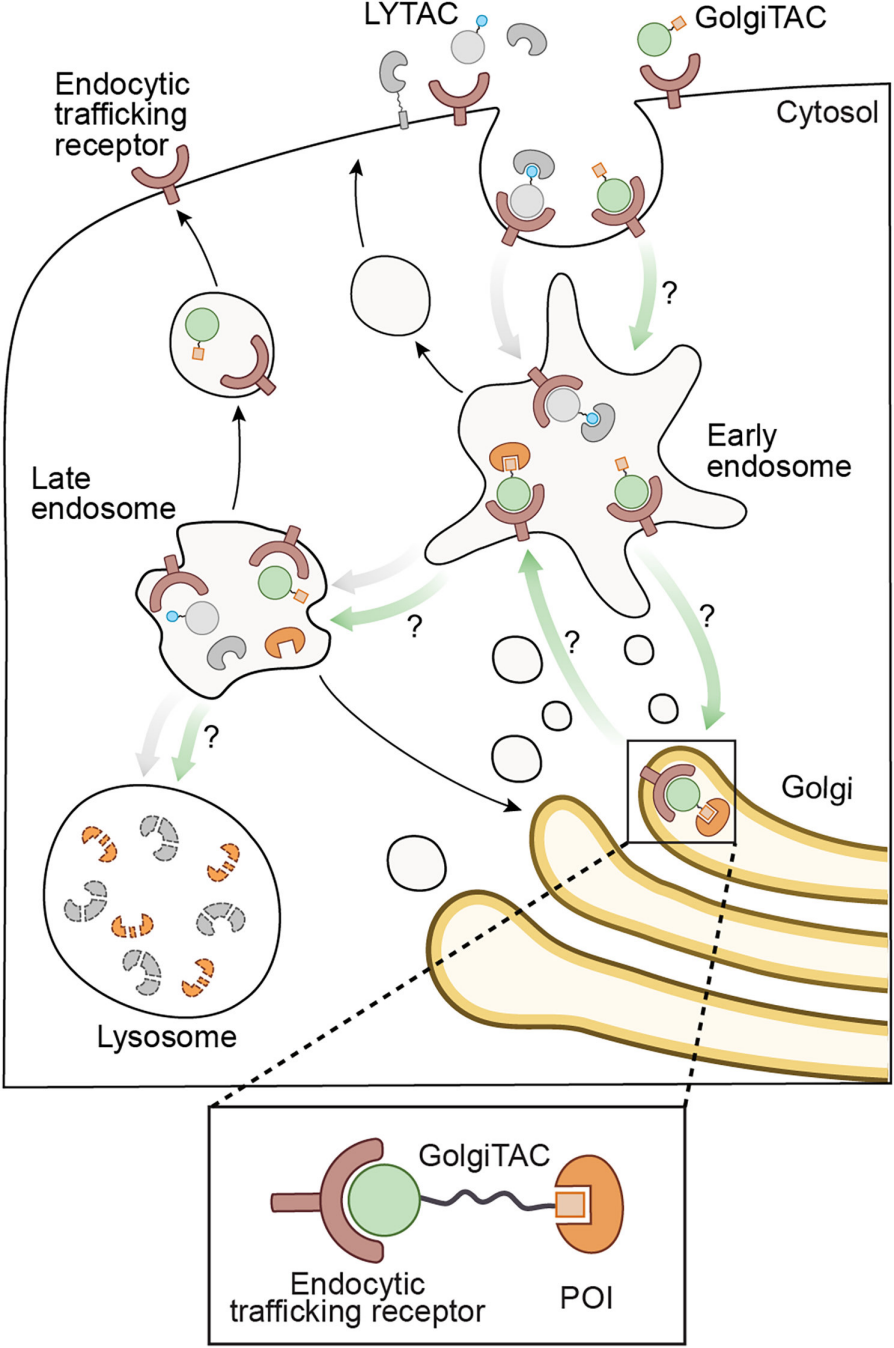
Proposed mechanism of GolgiTAC‐mediated degradation. Comparison of the established LYTAC pathway (gray arrows) with the proposed GolgiTAC pathway (green arrows). In the LYTAC modality, a LYTAC binds an extracellular protein of interest (POI) and recruits an endocytic trafficking receptor. The resulting ternary complex is internalized and trafficked through early and late endosomes. Acidification in the late endosome promotes dissociation of the receptor from the LYTAC–POI complex. The POI is then delivered to the lysosome for degradation, while the receptor can be recycled to the cell surface or Golgi. In the proposed GolgiTAC strategy, a GolgiTAC enters the cell via an endocytic trafficking receptor that cycles between the cell surface, endosomes, and Golgi. Upon binding a Golgi‐resident POI, the resulting complex could be redirected from the Golgi toward the endolysosomal pathway. Acidification in the late endosome might promote receptor dissociation, allowing the POI to be delivered to the lysosome for degradation, while the receptor and, potentially, the GolgiTAC are recycled to the Golgi and cell surface.

Although current LYTAC strategies have focused primarily on extracellular and plasma membrane‐associated proteins, the same conceptual framework could, in theory, be extended to intracellular trafficking pathways, particularly those connecting the Golgi apparatus, endosomes, and lysosomes. We term this proposed strategy Golgi‐targeting chimeras (GolgiTACs), which would extend receptor‐mediated lysosomal targeting to proteins residing in or transiting through the Golgi apparatus. Although GolgiTACs may exploit some of the same trafficking receptors used by LYTACs, including CI‐M6PR and sortilin, the critical distinction lies in the site of target engagement: whereas conventional LYTACs capture targets at the cell surface or in the extracellular space and promote their endocytosis, GolgiTACs would engage their targets within the Golgi apparatus and redirect them toward the endolysosomal system (Figure 4, green pathway).

Increasing mechanistic insights into cargo sorting and export from the Golgi apparatus have revealed how trafficking receptors orchestrate the selective delivery and recycling of proteins throughout the secretory pathway [19]. In the TGN, receptors such as CI‐M6PR and sortilin bind to cargo proteins and package them into transport carriers destined for endosomal compartments. From there, proteins are delivered to lysosomes, whereas the receptors dissociate from their ligands and recycle back to the Golgi apparatus or, in some cases, to the plasma membrane [19, 124].

Two M6P receptors mediate the intracellular sorting of lysosomal enzymes at the Golgi apparatus: the cation‐independent (CI‐M6PR) and the cation‐dependent mannose‐6‐phosphate receptor (CD‐M6PR). Although both contribute to lysosomal hydrolase trafficking, CI‐M6PR possesses a broader cargo repertoire and has become the principal receptor exploited for LYTACs. CI‐M6PR recognizes mannose‐6‐phosphate (M6P) glycans installed on newly synthesized lysosomal hydrolases in the TGN and packages them into clathrin‐coated transport vesicles destined for early endosomes [124, 125]. Cytosolic adaptor proteins, primarily the Golgi‐localized γ‐ear‐containing ARF‐binding proteins (GGAs) together with adaptor protein complex 1 (AP‐1), recognize sorting motifs within the cytosolic tail of CI‐M6PR and recruit clathrin, thereby coupling luminal cargo recognition to vesicle formation [126, 127, 128]. Upon arrival at the more acidic endosomal compartments, the lower pH promotes dissociation of the lysosomal hydrolases from CI‐M6PR, allowing the receptor to recycle back to the TGN, facilitated by the retromer complex, while the released hydrolases continue their transport to lysosomes [129]. This highly efficient recycling pathway has made CI‐M6PR the receptor of choice for most extracellular LYTAC strategies, despite the fact that only ~10% of the receptor population is present at the cell surface at the steady state [130]. Structurally, CI‐M6PR is a large type‐I transmembrane glycoprotein comprising a short cytosolic tail, a single transmembrane helix, and an extensive luminal ectodomain formed by 15 homologous repeats. Carbohydrate recognition is mainly mediated by four mannose receptor homology (MRH) domains. Domains 3 and 9 serve as the principal high‐affinity binding sites for mannose‐6‐phosphate (M6P), whereas domain 5 preferentially recognizes phosphodiester‐containing glycans [131, 132, 133, 134] (M6P‐GlcNAc) (Figure 5A). Recent studies show that domain 15 provides an additional, lower‐affinity binding site capable of recognizing both phosphomonoester and phosphodiester ligands, thereby broadening the receptor’s cargo repertoire. In addition to its carbohydrate‐binding sites, domain 11 binds insulin‐like growth factor 2 (IGF‐2), and domain 1 interacts with the urokinase plasminogen activator receptor (uPAR) and plasminogen (Plg) [134]. This architecture allows CI‐M6PR to interact with more than 60 endogenous lysosomal hydrolases (e.g., cathepsins, glycosidases, sulfatases, lipases, and nucleases) and several non‐M6P ligands [136]. Current LYTACs exploit this recognition mechanism by incorporating multivalent synthetic mannose‐6‐phosphonate glycopeptides (M6Pn). The phosphonate substitution renders these ligands resistant to phosphatase‐mediated hydrolysis, and although phosphonates have a higher second pKa than the corresponding phosphate esters (typically ~7.5 vs. ~6.3), they remain sufficiently ionized at physiological pH to preserve binding affinity for CI‐M6PR [137, 138]. Multivalent display further enhances receptor avidity and promotes efficient lysosomal delivery of antibodies, proteins, and other macromolecular cargos [6]. Recombinant or engineered IGF2‐based ligands, which bind a distinct site within domain 11 of CI‐M6PR, have also been explored for lysosomal targeting [139, 140].

**FIGURE 5:**
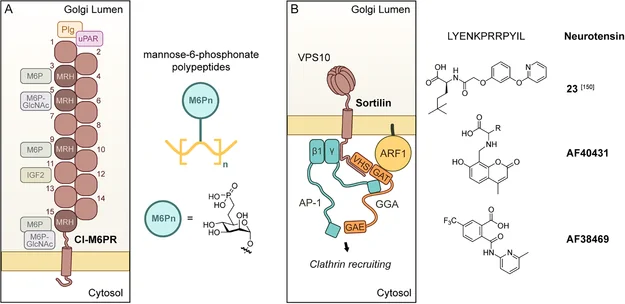
CI‐M6PR and sortilin structures and ligands. (A) CI‐M6PR comprises 15 luminal domains and a single transmembrane helix. Mannose receptor homology (MRH) domains 3, 9, and 15 bind mannose‐6‐phosphate (M6P), whereas domains 5 and 15 recognize phosphodiester‐glycans (M6P‐GlcNAc). Domain 1 binds the urokinase plasminogen activator receptor (uPAR) and plasminogen (Plg), and domain 11 interacts with IGF2R. Mannose‐6‐phosphonate polypeptide ligands are established ligands for targeting CI‐M6PR. (B) Sortilin is a type I transmembrane protein containing a luminal Vps10p‐type β‐propeller domain, a single transmembrane helix, and a short C‐terminal cytosolic tail containing sorting motifs recognized by Golgi‐localized γ‐ear‐containing ARF‐binding proteins (GGAs) and adaptor protein complex 1 (AP‐1). GGA interacts via its VHS domain with the C‐terminal part of the cytosolic sortilin tail. Its GGA and Tom1 (GAT) domains bind ADP‐ribosylation factor 1 (ARF1) at the membrane, while the hinge region can bind the γ‐ear domain of AP‐1. AP‐1 binds to sortilin at the N‐terminal part of the cytosolic tail. Clathrin is recruited by both GGA, via its γ‐adaptin ear (GAE) domain, and AP‐1, via the β1 hinge and appendage domains. These adaptor proteins recruit clathrin and induce vesicle formation. Neurotensin and several small molecules are established sortilin ligands [135].

Sortilin constitutes a complementary Golgi sorting receptor that recognizes structurally diverse protein cargos independently of glycosylation. As a member of the VPS10‐domain receptor family, sortilin binds numerous lysosomal enzymes, neuropeptides, lipoproteins, and membrane proteins, functioning both as an intracellular sorting receptor in the TGN and as an endocytic receptor at the plasma membrane [141, 142]. Similar to CI‐M6PR, sortilin recruits GGAs and AP‐1 through cytoplasmic sorting motifs to promote clathrin‐mediated transport toward endosomes before recycling back to the Golgi [141, 143] (Figure 5B). Unlike CI‐M6PR, however, sortilin relies on direct protein–protein recognition rather than carbohydrate‐based targeting, potentially providing greater flexibility for the design of synthetic trafficking ligands. Structurally, sortilin is a type I transmembrane receptor composed of a luminal VPS10 β‐propeller domain, a single transmembrane helix, and a short cytosolic tail containing multiple adaptor‐binding motifs that regulate intracellular trafficking [142, 144] (Figure 5B). The VPS10 domain forms a large ligand‐binding cavity capable of accommodating chemically and structurally diverse cargos through multiple partially overlapping binding surfaces. Structural studies further suggest that sortilin undergoes pH‐dependent conformational changes and reversible dimerization during endosomal trafficking [145, 146, 147]. Dimerization is associated with the collapse of the ligand‐binding pocket and reduced ligand accessibility, thereby promoting cargo release prior to receptor recycling. However, the physiological role of sortilin dimerization, how it is regulated, and the extent to which it directly controls ligand binding *in vivo* remain subjects of ongoing debate [146, 147].

Unlike CI‐M6PR, whose ligand repertoire is largely defined by mannose‐6‐phosphate glycans, sortilin recognizes peptide sorting motifs found in numerous endogenous cargos, including lysosomal hydrolases such as prosaposin, cathepsins, and acid sphingomyelinase, as well as neurotrophic factors, apolipoproteins, and lipoprotein‐associated proteins [142, 148]. This broad substrate specificity has made sortilin a central regulator of lysosomal enzyme delivery, lipid metabolism, neuronal signaling, and secretory protein trafficking. To date, the neuropeptide neurotensin is the best‐characterized sortilin ligand and binds within the central tunnel of the VPS10 domain, where its C‐terminal residues establish the principal receptor interactions, as revealed by crystallographic studies [149]. This binding pocket has subsequently served as the basis for the development of the first selective small‐molecule sortilin ligands, including **AF40431** [150] and the orally bioavailable derivative **AF38469** [151], which mimic key neurotensin interactions while competing for the same binding site [142, 152] (Figure 5B). More recently, neurotensin‐derived peptides and small‐molecule ligand **23** [135] have also been incorporated into LYTACs, demonstrating that sortilin can be pharmacologically recruited to direct extracellular cargo toward lysosomal degradation [113, 114]. Although sortilin has so far been explored to a much lesser extent than CI‐M6PR for lysosome‐targeting applications, its biology offers several attractive features for Golgi‐directed degradation strategies. Its prominent localization within the TGN and endosomal system, together with its ability to recognize structurally diverse protein cargos and small molecules, makes it a complementary receptor platform for manipulating Golgi‐to‐lysosome trafficking.

In addition to CI‐M6PR and sortilin, lysosomal trafficking also relies on alternative sorting receptors. For example, LIMP‐2 (SCARB2) mediates the M6P‐independent transport of glucocerebrosidase (GBA1) from the Golgi apparatus to lysosomes [152], illustrating that multiple endogenous receptor systems exist for lysosomal delivery. This highly dynamic endosome‐Golgi‐lysosome axis provides an attractive opportunity to intercept Golgi‐resident proteins and proteins trafficking through the Golgi and reroute them away from their physiological destination toward degradation. An emerging question is whether endogenous sorting receptors could be hijacked to alter the trafficking fate of selected Golgi proteins. Although GolgiTACs have not yet been developed, we envision that such an approach could provide a conceptual framework for achieving this goal (Figure 4, green pathway). In contrast to conventional targeted protein degraders, GolgiTACs would not only require high‐affinity binding to the target protein but also the ability to encode specific intracellular trafficking instructions, enabling the target to be rerouted from the secretory pathway toward lysosomal degradation. For example, ligands could be designed to recruit receptors that promote export from the Golgi apparatus, suppress retrograde recycling, and/or favor progression toward late endosomes and lysosomes. In this way, receptor engagement would become an active routing decision rather than a passive binding event, expanding TPD beyond inducing trimeric complex formation to the control of intracellular trafficking.

Many challenges remain before such Golgi‐to‐lysosome targeting strategies become a reality. For potential GolgiTACs, a central unresolved question is whether receptor engagement alone will be sufficient to reroute target proteins or whether these GolgiTACs must actively bias intracellular trafficking by promoting export from the Golgi apparatus. Whereas some Golgi‐transiting cargos may simply piggy‐back on the constitutive trafficking itinerary of endogenous Golgi sorting receptors, many Golgi‐resident proteins are maintained by retention and retrieval mechanisms that could limit their accessibility to this potential TPD modality. Whether receptor tethering alone is sufficient or whether GolgiTACs must actively overcome these retention pathways is likely to depend on the intrinsic trafficking behavior of the target protein and remains an important open question.

Receptor‐mediated sorting depends on binding affinity but also on where and when receptor binding and dissociation occur during intracellular transport. Luminal pH gradually decreases along the secretory pathway, from the Golgi apparatus (approximately pH 6.5–6.7) to early endosomes (pH 6.0–6.5), late endosomes (pH 5.0–6.0), and lysosomes (pH 4.5–5.0), profoundly influencing receptor‐cargo interactions. Conventional LYTACs are not designed for efficient recycling of the degrader after target internalization, limiting their ability to undergo repeated rounds of target degradation. To overcome this limitation, Lycia Therapeutics has developed catalytic LYTACs (cataLYTACs), which incorporate pH‐sensitive target binding to promote target release within the acidic endosomal environment while enabling the recycling of the cataLYTAC to the surface. One example is **LCA‐0061**, an IgE‐targeting cataLYTAC currently being developed for allergic diseases [153]. Intracellular GolgiTAC molecules may require more finely tuned pH‐dependent binding properties than current extracellular LYTACs, allowing stable receptor engagement in the Golgi or early endosomes while promoting cargo release in late endosomes or lysosomes to enable efficient degradation and receptor recycling. Ligand pKa values, receptor‐ and target‐binding kinetics, valency, avidity, and subcellular accessibility are therefore likely to become critical design parameters. The balance between lysosomal delivery and competing recycling pathways represents an additional consideration. Ahn et al. demonstrated that retromer‐dependent recycling of LYTAC‐CI‐M6PR complexes from endosomes back to the plasma membrane can compete with their delivery to lysosomes, with disruption of this pathway enhancing LYTAC‐mediated degradation. Moreover, competition with endogenous receptor cargo, such as M6P‐modified proteins in the case of CI‐M6PR, may influence receptor availability and consequently GolgiTAC efficiency [154].

Additional complexity arises from the biological function of the trafficking receptors themselves. These receptors differ in tissue distribution, subcellular localization, recycling dynamics, and endogenous cargo load. Competition with natural ligands may reduce degradation efficiency while simultaneously perturbing essential trafficking pathways. Excessive or prolonged engagement of receptors such as CI‐M6PR or sortilin could impair lysosomal enzyme delivery, lipid homeostasis, or neuronal protein trafficking, potentially resulting in pathological consequences.

An additional requirement for expanding GolgiTPD will be the development of a broader repertoire of ligands for intracellular trafficking receptors. Beyond exploiting known endogenous ligands, established drug‐discovery approaches, including conventional high‐throughput screening and affinity‐selection technologies using DNA‐encoded, display‐based, or synthetic combinatorial libraries, could provide new receptor binders [155, 156]. Importantly, there is already precedent for this strategy: the small‐molecule sortilin ligand **AF40431** was identified through high‐throughput screening using a radioligand binding assay and subsequently validated through structure–activity relationship studies and structural characterization [150]. The application of similar approaches to CI‐M6PR will depend on the development of robust and scalable receptor‐binding assays. This may be particularly challenging given its multidomain architecture and the presence of several ligand‐binding sites with distinct recognition properties [157].

Although substantial challenges remain, the progress in understanding Golgi sorting mechanisms provides an increasingly detailed blueprint for manipulating intracellular trafficking. Rather than simply recruiting degradative machinery, future Golgi‐directed degraders may encode trafficking information that actively determines where proteins travel within the secretory pathway. By combining target recognition with selective engagement of endogenous Golgi sorting receptors and trafficking adaptors, it may become possible to redirect Golgi‐resident or Golgi‐transiting proteins toward lysosomal destruction while preserving physiological receptor recycling. Such strategies would represent a conceptual shift in TPD.

## 6 Conclusions and Future Directions in Golgi‐TPD

The Golgi apparatus, at the center of the secretory pathway, provides an ideal platform for TPD strategies accessing secretory and transmembrane proteins. Proteins enter the Golgi from the ER during the production phase, while numerous proteins from the plasma membrane cycle back to the Golgi via the endosomal system. Consequently, the Golgi apparatus provides access to a large substrate pool comprising Golgi‐resident proteins, secretory cargo, and recycling membrane proteins. At the same time, the dynamic nature of the Golgi apparatus and its sensitivity to stress present unique challenges for the development of TPD strategies.

The current and emerging strategies for Golgi‐TPD are summarized in Table 1. These approaches span distinct degradation mechanisms and target classes, ranging from broad Golgi protein depletion and autophagic degradation at the organelle level to selective proteasomal degradation and the proposed receptor‐mediated rerouting of Golgi proteins to lysosomes. A better understanding of TM‐E3 ligases holds the potential to expand ubiquitin‐dependent modalities at the Golgi. For these modalities, the type of ubiquitin signal presents an important point of refinement as it strongly contributes to the fate of the target. It may control substrate targeting to lysosomes versus the proteasome and possibly induce ubiquitin‐dependent autophagy through ubiquitin‐binding cargo receptors. Recent studies of receptor‐mediated Golgiphagy have provided mechanistic insight into bulk Golgi turnover. We propose that integrating these insights with emerging autophagy‐inducing approaches may provide a way forward to developing Golgiphagy‐inducing strategies and eventually to finetuning their substrate specificity. Eventually, hijacking endogenous receptor‐mediated Golgi‐to‐lysosome trafficking may provide the conceptual basis for the future development of GolgiTACs, intracellular degradation molecules conceptually related to extracellular LYTACs. Realizing such strategies will require precise spatial and temporal control of receptor engagement together with a deeper understanding of receptor recycling, cargo release, and Golgi‐to‐lysosome trafficking.

**TABLE 1:** Current and emerging strategies for targeted protein degradation at the Golgi apparatus.

Golgi TPD strategy	Targets	Mechanism of action	Key cellular machinery	Current challenges and limitations
Golgi‐disrupting small‐molecule stressors	Potential targets: Golgi proteins dependent on intact Golgi architecture and lipid/pH homeostasis. Reported targets: including QSOX2, GOLIM4, GALNT2, SLC35B2, SDF4, GLG1, and KIAA2013.	Ionophores (monensin, nigericin, salinomycin) and OSBP inhibitors (OSW1, oxybypin, orpinolide) pharmacologically alter Golgi pH and lipid composition, causing Golgi fragmentation and remodeling, including depletion of a subset of Golgi proteins.	Golgi‐endosome interface	Indirect and nonspecific; potential cytotoxicity and disruption of overall Golgi integrity
Autophagy‐targeting chimeras	Potential targets: Golgi membrane and Golgi fragments, containing transiting secretory and/or transmembrane cargo proteins.	Recruitment of the autophagy machinery to induce Golgiphagy or GOMED.	YIPF3/4, TM9SF3, Optineurin, p62, TAX1BP1	Limited specificity; LC3 lipidation may induce cellular stress responses; potential impact on Golgi integrity
PROTACs	Potential targets: Peripheral and transmembrane Golgi proteins, as well as transmembrane cargo proteins, with an accessible and ligandable cytosolic domain. Reported targets: To date limited to engineered/fusion protein systems: • AID system: RAB1B, GRASP55, GRASP65, and COG4 • Hydrophobic tagging of HaloTag2 (HyT36): GRASP55 and TMEM115 • dTAG system (dTAG7, dTAG13): SLC35B2, SLC38A2	Induced proximity to an E3 ligase promotes substrate ubiquitination, followed by protein extraction from the Golgi membrane where required and proteasomal degradation. Potential adaptations include classical PROTACs, proposed AbTAC‐like molecules, or recruitment of Golgi‐localized transmembrane E3 ligases.	E3‐ligase, ubiquitination machinery, p97/VCP, proteasome	Limited repertoire of characterized Golgi‐associated E3 ligases; target requires an accessible ligandable cytosolic domain and suitable topology for ubiquitination and, for transmembrane proteins, extraction
Proposed GolgiTACs	Potential targets: Soluble luminal proteins and transmembrane proteins with an accessible Golgi‐luminal domain, including proteins transiting through the Golgi. Reported targets: N/A (No validated molecular example to date)	A bifunctional molecule links a luminal Golgi target protein to a trafficking receptor, hijacking Golgi‐endosome‐lysosome transport pathways to promote lysosomal delivery and degradation.	Sortilin, CI‐M6PR, endosomal sorting machinery, lysosome	Complex anterograde and retrograde trafficking; pH‐dependent ligand‐receptor interactions; competition with endogenous ligands; requirement for an accessible luminal target epitope

From the substrate’s perspective, the domain architecture and topology of the target protein should be considered primary determinants when designing a Golgi‐TPD modality. While classical PROTACs require a ligandable cytosolic domain and accessible cytosolic lysine residues, GolgiTAC molecules will rely on engaging luminal domains and could be particularly suited to secretory proteins and Golgi transmembrane proteins with large luminal domains. To cover the breadth and diversity of target proteins at the Golgi apparatus, it will be critical to continue developing complementary TPD strategies.

Finally, we would like to highlight that in the design of Golgi‐TPD strategies, potential bystander effects arising from perturbation of Golgi homeostasis should be carefully considered. Small‐molecule Golgi stressors have demonstrated that chemically‐induced Golgi protein depletion is feasible but, conversely, also illustrate that Golgi‐TPD strategies may trigger stress responses and/or changes in Golgi morphology. Golgi stressors in their current form may therefore be limited to cell biology studies rather than therapeutic applications. Similarly, excessive engagement of endogenous trafficking receptors such as sortilin or CI‐M6PR by GolgiTACs may disturb the physiological balance between incoming and outgoing Golgi traffic. Unwanted effects may include the disruption of lysosomal function due to competition with lysosomal enzyme transport. Equally, inducing post‐translational modifications at the Golgi apparatus, such as ubiquitination or LC3 lipidation, may drive cells into a stressed state and activate stress response pathways. While these responses may be favorable to the intended cellular effects of certain Golgi‐TPD strategies, they may have to be controlled in other instances, posing an additional design aspect to consider. Proof‐of‐concept studies in cell culture will assess these effects, enabling their characterization and resolution, before progressing with Golgi‐TPD strategies as therapeutic modalities.

In summary, the field of Golgi‐targeted degradation currently spans from broad Golgi stressors to emerging, conceptually diverse, Golgi‐TPD strategies. Collectively, these strategies show great potential to effectively expand TPD toward a large fraction of the secretory and transmembrane proteome, which remains largely inaccessible to current degradation strategies. Integrating recent advances in Golgi‐related intracellular trafficking, protein quality control, and protein degradation mechanisms, as well as targeted degradation chemistries, will undoubtedly accelerate the development of selective and effective Golgi‐directed degraders.

## Disclaimer

The opinions expressed in this publication are the view of the author(s) and do not necessarily reflect the opinions or views of ChemBioChem, the Publisher, ChemistryEurope, or the affiliated editors.

## Funding

This study was supported by the European Research Council (Grant ERC‐2024‐STG 101163762 SWEET DEGRADATION) and the Deutsche Forschungsgemeinschaft (Grant DFG Project number 577589279).

## Conflicts of Interest

The authors declare no conflicts of interest.

## Supporting information

The authors have cited additional references within the Supporting Information [158, 159, 160, 161, 162, 163, 164].

## Data Availability

The data supporting the Golgi proteome are available in the Supporting Information of this article.

## References

[1] M. Békés , D. R. Langley , and C. M. Crews , “PROTAC Targeted Protein Degraders: The Past Is Prologue,” Nature Reviews Drug Discovery 21 (2022): 181–200, 10.1038/s41573-021-00371-6. PMC8765495 35042991

[2] K. M. Sakamoto , K. B. Kim , A. Kumagai , F. Mercurio , C. M. Crews , and R. J. Deshaies , “Protacs: Chimeric Molecules that Target Proteins to the Skp1‐Cullin‐F Box Complex for Ubiquitination and Degradation,” Proceedings of the National Academy of Sciences of the United States of America 98 (2001): 8554–8559, 10.1073/pnas.141230798. PMC37474 11438690

[3] A. Mullard , “First PROTAC Gains FDA Approval, Bolstering Targeted Protein Degradation and Induced Proximity Ambitions,” Nature Reviews Drug Discovery 25 (2026): 411–411, 10.1038/d41573-026-00078-6. 42103863

[4] M. Konstantinidou and M. R. Arkin , “Molecular Glues for Protein‐Protein Interactions: Progressing Toward a New Dream,” Cell Chemical Biology 31 (2024): 1064–1088, 10.1016/j.chembiol.2024.04.002. PMC11193649 38701786

[5] S. L. Schreiber , “The Rise of Molecular Glues,” Cell 184 (2021): 3–9, 10.1016/j.cell.2020.12.020. 33417864

[6] S. M. Banik , K. Pedram , S. Wisnovsky , G. Ahn , N. M. Riley , and C. R. Bertozzi , “Lysosome‐Targeting Chimaeras for Degradation of Extracellular Proteins,” Nature 584 (2020): 291–297, 10.1038/s41586-020-2545-9. PMC7727926 32728216

[7] G. Ahn , S. M. Banik , C. L. Miller , N. M. Riley , J. R. Cochran , and C. R. Bertozzi , “LYTACs that Engage the Asialoglycoprotein Receptor for Targeted Protein Degradation,” Nature Chemical Biology 17 (2021): 937–946, 10.1038/s41589-021-00770-1. PMC8387313 33767387

[8] B. Niebel , W. Chen , K. Mukai , and C. Henry , “LYTACs and Other Extracellular Targeted Protein Degradation TACnologies: Guiding Principles and Potential Clinical Prospects,” Drug Discovery Today 31 (2026): 104601, 10.1016/j.drudis.2026.104601. 41534633

[9] D. Takahashi and H. Arimoto , “Selective Autophagy as the Basis of Autophagy‐Based Degraders,” Cell Chemical Biology 28 (2021): 1061–1071, 10.1016/j.chembiol.2021.05.006. 34087173

[10] D. Takahashi , J. Moriyama , T. Nakamura , et al., “AUTACs: Cargo‐Specific Degraders Using Selective Autophagy,” Molecular Cell 76 (2019): 797–810.e10, 10.1016/j.molcel.2019.09.009. 31606272

[11] C. H. Ji , H. Y. Kim , M. J. Lee , et al., “The AUTOTAC Chemical Biology Platform for Targeted Protein Degradation via the Autophagy‐Lysosome System,” Nature Communications 13 (2022): 904, 10.1038/s41467-022-28520-4. PMC8850458 35173167

[12] Z. Li , C. Zhu , Y. Ding , Y. Fei , and B. Lu , “ATTEC: A Potential New Approach to Target Proteinopathies,” Autophagy 16 (2020): 185–187, 10.1080/15548627.2019.1688556. PMC6984452 31690177

[13] M. P. Schwalm , J. Dopfer , A. Kumar , et al., “Critical Assessment of LC3/GABARAP Ligands Used for Degrader Development and Ligandability of LC3/GABARAP Binding Pockets,” Nature Communications 15 (2024): 10204, 10.1038/s41467-024-54409-5. PMC11589570 39587067

[14] Z. Li , C. Wang , Z. Wang , et al., “Allele‐Selective Lowering of Mutant HTT Protein by HTT–LC3 Linker Compounds,” Nature 575 (2019): 203–209, 10.1038/s41586-019-1722-1. 31666698

[15] Y. Fu , N. Chen , Z. Wang , S. Luo , Y. Ding , and B. Lu , “Degradation of Lipid Droplets by Chimeric Autophagy‐Tethering Compounds,” Cell Research 31, no. 9 (2021): 965–979, 10.1038/s41422-021-00532-7. PMC8410765 34239073

[16] W. W. Y. Yim and N. Mizushima , “Lysosome Biology in Autophagy,” Cell Discovery 6 (2020): 6, 10.1038/s41421-020-0141-7. PMC7010707 32047650

[17] L. Zhao , J. Zhao , K. Zhong , A. Tong , and D. Jia , “Targeted Protein Degradation: Mechanisms, Strategies and Application,” Signal Transduction and Targeted Therapy 7 (2022): 113, 10.1038/s41392-022-00966-4. PMC8977435 35379777

[18] J. Parolek , Y. Kim , and C. G. Burd , “Lipid Metabolism and Trafficking in the Golgi Apparatus,” Sub‐Cellular Biochemistry 111 (2026): 177–194, 10.1007/978-3-032-16833-7_8. 41718977

[19] E. T. Watson , W. R. Wegeng , S. Aravani , A. M. Ernst , and J. von Blume , “Mechanistic Insights Into Cargo Sorting and Export From the Golgi Apparatus,” Nature Reviews Molecular Cell Biology 26 (2025): 940–956, 10.1038/s41580-025-00907-3. PMC13208668 41214333

[20] P. A. Gleeson , “Impact of Golgi Dynamics and Golgi Morphology on the Regulation of Glycosylation,” Biochimica et Biophysica Acta (BBA)—General Subjects 1870 (2026): 130919, 10.1016/j.bbagen.2026.130919. 41713215

[21] H. H. Freeze , M. Boyce , N. E. Zachara , G. W. Hart , and R. L. Schnaar , “Glycosylation Precursors,” in Essentials of Glycobiology, chap. 5, 4th ed., eds. A. Varki , R. D. Cummings , J. D. Esko (Cold Spring Harbor Laboratory Press, 2022), 10.1101/glycobiology.4e.5. 35536946

[22] P. Stanley , “Golgi Glycosylation,” Cold Spring Harbor Perspectives in Biology 3 (2011): a005199, 10.1101/cshperspect.a005199. PMC3062213 21441588

[23] K. W. Moremen , M. Tiemeyer , and A. V. Nairn , “Vertebrate Protein Glycosylation: Diversity, Synthesis and Function,” Nature Reviews Molecular Cell Biology 13, no. 7 (2012): 448–462, 10.1038/nrm3383. PMC3934011 22722607

[24] C. L. de Graffenried and C. R. Bertozzi , “Golgi Localization of Carbohydrate Sulfotransferases Is a Determinant of L‐Selectin Ligand Biosynthesis,” Journal of Biological Chemistry 278 (2003): 40282–40295, 10.1074/jbc.M304928200. 12855678

[25] K. G. Bowman and C. R. Bertozzi , “Carbohydrate Sulfotransferases: Mediators of Extracellular Communication,” Chemistry & Biology 6 (1999): R9–R22, 10.1016/S1074-5521(99)80014-3. 9889154

[26] V. A. Bankaitis , R. Garcia‐Mata , and C. J. Mousley , “Golgi Membrane Dynamics and Lipid Metabolism,” Current Biology 22 (2012): R414–R424, 10.1016/j.cub.2012.03.004. PMC4059015 22625862

[27] D. Demaegd , F. Foulquier , A.‐S. Colinet , et al., “Newly Characterized Golgi‐Localized Family of Proteins Is Involved in Calcium and pH Homeostasis in Yeast and Human Cells,” Proceedings of the National Academy of Sciences 110 (2013): 6859–6864, 10.1073/pnas.1219871110. PMC3637739 23569283

[28] A. K. Gillingham and S. Munro , “Finding the Golgi: Golgin Coiled‐Coil Proteins Show the Way,” Trends in Cell Biology 26, no. 6 (2016): 399–408, 10.1016/j.tcb.2016.02.005. 26972448

[29] J. Malsam and T. H. Söllner , “Organization of SNAREs Within the Golgi Stack,” Cold Spring Harbor Perspectives in Biology 3 (2011): a005249, 10.1101/cshperspect.a005249. PMC3179334 21768609

[30] C. Makhoul , P. Gosavi , and P. A. Gleeson , “The Golgi Architecture and Cell Sensing,” Biochemical Society Transactions 46 (2018): 1063–1072, 10.1042/BST20180323. 30242119

[31] U. Rein , U. Andag , R. Duden , H. D. Schmitt , and A. Spang , “ARF‐GAP–Mediated Interaction Between the ER‐Golgi v‐SNAREs and the COPI Coat,” Journal of Cell Biology 157 (2002): 395–404, 10.1083/jcb.200112092. PMC2173288 11970962

[32] E. Sztul and V. Lupashin , “Role of Vesicle Tethering Factors in the ER–Golgi Membrane Traffic,” FEBS Letters 583 (2009): 3770–3783, 10.1016/j.febslet.2009.10.083. PMC2788073 19887069

[33] L. L. Thomas and J. C. Fromme , “Extensive GTPase Crosstalk Regulates Golgi Trafficking and Maturation,” Current Opinion in Cell Biology 65 (2020): 1–7, 10.1016/j.ceb.2020.01.014. PMC7483271 32143122

[34] D. K. Banfield , “Mechanisms of Protein Retention in the Golgi,” Cold Spring Harbor Perspectives in Biology 3 (2011): a005264–a005264, 10.1101/cshperspect.a005264. PMC3140682 21525512

[35] F. Zappa , M. Failli , and M. A. De Matteis , “The Golgi Complex in Disease and Therapy,” Current Opinion in Cell Biology 50 (2018): 102–116, 10.1016/j.ceb.2018.03.005. 29614425

[36] K. Legler , R. Rosprim , T. Karius , et al., “Reduced Mannosidase MAN1A1 Expression Leads to Aberrant N‐Glycosylation and Impaired Survival in Breast Cancer,” British Journal of Cancer 118, no. 6 (2018): 847–856, 10.1038/bjc.2017.472. PMC5877434 29381688

[37] A. Nashed , K. Dilsook , T. Senapathi , and K. J. Naidoo , “An In Vitro Approach for Simulating Divergent Golgi O‐Glycosylation of Tumor‐Associated MUC1 From Normal MUC1,” Nature Communications 17, no. 1 (2026): 3619, 10.1038/s41467-026-72151-y. PMC13103387 42020444

[38] X. Xu , Q. Peng , X. Jiang , et al., “Altered Glycosylation in Cancer: Molecular Functions and Therapeutic Potential,” Cancer Communications 44 (2024): 1316–1336, 10.1002/cac2.12610. PMC11570773 39305520

[39] S. S. Pinho and C. A. Reis , “Glycosylation in Cancer: Mechanisms and Clinical Implications,” Nature Reviews Cancer 15, no. 9 (2015): 540–555, 10.1038/nrc3982. 26289314

[40] S. R. Akaaboune and Y. Wang , “Golgi Defect as a Major Contributor to Lysosomal Dysfunction,” Frontiers in Cell and Developmental Biology 12 (2024): 1386149, 10.3389/fcell.2024.1386149. PMC11076776 38721528

[41] J. Zhang , A. Kennedy , D. M. de Melo Jorge , et al., “SARS‐CoV‐2 Remodels the Golgi Apparatus to Facilitate Viral Assembly and Secretion,” PLOS Pathogens 21 (2025): e1013295, 10.1371/journal.ppat.1013295. PMC12208438 40540531

[42] A. Iannuzzo , S. Delafontaine , R. El Masri , et al., “Autoinflammatory Patients With Golgi‐Trapped CDC42 Exhibit Intracellular Trafficking Defects Leading to STING Hyperactivation and ER Stress,” Nature Communications 15 (2024): 9940, 10.1038/s41467-024-54294-y. PMC11569173 39550374

[43] L. Fourriere and P. A. Gleeson , “The Dynamic Role of the Golgi Apparatus in Neuronal Function and Alzheimer’s Disease,” in Subcellular Biochemistry, vol. 111 (Springer, 2026), 441–462, 10.1007/978-3-032-16833-7_18. 41718987

[44] C. Makhoul , P. Gosavi , and P. A. Gleeson , “Golgi Dynamics: The Morphology of the Mammalian Golgi Apparatus in Health and Disease,” Frontiers in Cell and Developmental Biology 7 (2019): 112, 10.3389/fcell.2019.00112. PMC6616279 31334231

[45] R. Natarajan and A. D. Linstedt , “A Cycling Cis‐Golgi Protein Mediates Endosome‐to‐Golgi Traffic,” Molecular Biology of the Cell 15 (2004): 4798–4806, 10.1091/mbc.e04-05-0366. PMC524728 15331763

[46] S. Mukhopadhyay and A. D. Linstedt , “Manganese Blocks Intracellular Trafficking of Shiga Toxin and Protects Against Shiga Toxicosis,” Science 335 (2012): 332–335, 10.1126/science.1215930. PMC5367627 22267811

[47] S. Venkat and A. D. Linstedt , “Manganese‐Induced Trafficking and Turnover of GPP130 Is Mediated by Sortilin,” Molecular Biology of the Cell 28 (2017): 2569–2578, 10.1091/mbc.e17-05-0326. PMC5597328 28768823

[48] R. Tewari , T. Jarvela , and A. D. Linstedt , “Manganese Induces Oligomerization to Promote Down‐Regulation of the Intracellular Trafficking Receptor Used by Shiga Toxin,” Molecular Biology of the Cell 25 (2014): 3049–3058, 10.1091/mbc.e14-05-1003. PMC4230593 25079690

[49] C. Bachert , T. H. Lee , and A. D. Linstedt , “Lumenal Endosomal and Golgi‐Retrieval Determinants Involved in pH‐Sensitive Targeting of an Early Golgi Protein,” Molecular Biology of the Cell 12 (2001): 3152–3160, 10.1091/mbc.12.10.3152. PMC60163 11598199

[50] S. Puri , C. Bachert , C. J. Fimmel , and A. D. Linstedt , “Cycling of Early Golgi Proteins via the Cell Surface and Endosomes Upon Lumenal pH Disruption,” Traffic 3 (2002): 641–653, 10.1034/j.1600-0854.2002.30906.x. 12191016

[51] X. Tan , D. L. Cardin , S. Wang , Y. Xu , and W. K. Russell , “Monensin Suppresses EMT‐Driven Cancer Cell Motility by Inducing Golgi pH–Dependent Exocytosis of GOLIM4,” Proceedings of the National Academy of Sciences 122 (2025): e2501347122, 10.1073/pnas.2501347122. PMC12280883 40632561

[52] G. Simon , S. Abraham Pol , M. Dendusic , et al., “Stress‐Induced Proteome Remodeling at the Golgi–Endosome Interface,” Journal of Cell Science 139 (2026): jcs264535, 10.1242/jcs.264535. PMC13245918 41989390

[53] N. He , L. Depta , C. Rossetti , et al., “Inhibition of OSBP Blocks Retrograde Trafficking by Inducing Partial Golgi Degradation,” Nature Chemical Biology 21 (2025): 203–214, 10.1038/s41589-024-01653-x. 38907112

[54] M. Cigler , H. Imrichova , F. Frommelt , et al., “Orpinolide Disrupts a Leukemic Dependency on Cholesterol Transport by Inhibiting OSBP,” Nature Chemical Biology 21 (2025): 193–202, 10.1038/s41589-024-01614-4. PMC11782089 38907113

[55] M. Marjanović , A.‐M. Mikecin Dražić , M. Mioč , et al., “Salinomycin Disturbs Golgi Function and Specifically Affects Cells in Epithelial‐to‐Mesenchymal Transition,” Journal of Cell Science 136 (2023): jcs260934, 10.1242/jcs.260934. 37545292

[56] Z. Zhang , R. Venditti , L. Ran , et al., “Distinct Changes in Endosomal Composition Promote NLRP3 Inflammasome Activation,” Nature Immunology 24 (2023): 30–41, 10.1038/s41590-022-01355-3. PMC9810532 36443515

[57] M. Podinovskaia , C. Prescianotto‐Baschong , D. P. Buser , and A. Spang , “A Novel Live‐Cell Imaging Assay Reveals Regulation of Endosome Maturation,” eLife 10 (2021): e70982, 10.7554/eLife.70982. PMC8635980 34846303

[58] H. H. Mollenhauer , D. J. Morré , and L. D. Rowe , “Alteration of Intracellular Traffic by Monensin; Mechanism, Specificity and Relationship to Toxicity,” Biochimica et Biophysica Acta 1031 (1990): 225–246, 10.1016/0304-4157(90)90008-z. PMC7148783 2160275

[59] Y. V. Serebrenik , D. Hellerschmied , M. Toure , F. López‐Giráldez , D. Brookner , and C. M. Crews , “Targeted Protein Unfolding Uncovers a Golgi‐Specific Transcriptional Stress Response,” Molecular Biology of the Cell 29 (2018): 1284–1298, 10.1091/mbc.E17-11-0693. PMC5994893 29851555

[60] B. Mesmin , J. Bigay , J. Polidori , D. Jamecna , S. Lacas‐Gervais , and B. Antonny , “Sterol Transfer, PI4P Consumption, and Control of Membrane Lipid Order by Endogenous OSBP,” EMBO Journal 36 (2017): 3156–3174, 10.15252/embj.201796687. PMC5666618 28978670

[61] M. A. De Matteis , M. Fico , and R. Venditti , “Regulation and Function of PI4P at the Golgi Complex,” Biochimica et Biophysica Acta (BBA)‍—‍Molecular and Cell Biology of Lipids 1870 (2025): 159626, 10.1016/j.bbalip.2025.159626. 40350028

[62] A. W. G. Burgett , T. B. Poulsen , K. Wangkanont , et al., “Natural Products Reveal Cancer Cell Dependence on Oxysterol‐Binding Proteins,” Nature Chemical Biology 7 (2011): 639–647, 10.1038/nchembio.625. PMC3158287 21822274

[63] K. Oh‐hashi , H. Nakamura , H. Ogawa , Y. Hirata , and K. Sakurai , “Elucidation of OSW‐1‐Induced Stress Responses in Neuro2a Cells,” International Journal of Molecular Sciences 24 (2023): 5787, 10.3390/ijms24065787. PMC10059990 36982858

[64] A. Bensimon , M. D. Pizzagalli , F. Kartnig , et al., “Targeted Degradation of SLC Transporters Reveals Amenability of Multi‐Pass Transmembrane Proteins to Ligand‐Induced Proteolysis,” Cell Chemical Biology 27 (2020): 728–739.e9, 10.1016/j.chembiol.2020.04.003. PMC7303955 32386596

[65] Y. Chen , Y. Wu , X. Tian , G. Shao , Q. Lin , and A. Sun , “Golgiphagy: A Novel Selective Autophagy to the fore,” Cell & Bioscience 14 (2024): 130, 10.1186/s13578-024-01311-8. PMC11495120 39438975

[66] J. N. S. Vargas , M. Hamasaki , T. Kawabata , R. J. Youle , and T. Yoshimori , “The Mechanisms and Roles of Selective Autophagy in Mammals,” Nature Reviews Molecular Cell Biology 24 (2023): 167–185, 10.1038/s41580-022-00542-2. 36302887

[67] Y. Feng , D. He , Z. Yao , and D. J. Klionsky , “The Machinery of Macroautophagy,” Cell Research 24 (2014): 24–41, 10.1038/cr.2013.168. PMC3879710 24366339

[68] J.‐C. Farré and S. Subramani , “Mechanistic Insights Into Selective Autophagy Pathways: Lessons From Yeast,” Nature Reviews Molecular Cell Biology 17 (2016): 537–552, 10.1038/nrm.2016.74. PMC5549613 27381245

[69] T. Kotani and H. Nakatogawa , “Core Principles of Autophagy Initiation Mechanisms,” Nature Structural & Molecular Biology 33 (2026): 408–419, 10.1038/s41594-026-01752-4. 41721018

[70] K. L. Hickey , S. Swarup , I. R. Smith , et al., “Proteome Census Upon Nutrient Stress Reveals Golgiphagy Membrane Receptors,” Nature 623 (2023): 167–174, 10.1038/s41586-023-06657-6. PMC10620096 37757899

[71] S. Kitta , T. Kaminishi , M. Higashi , et al., “YIPF3 and YIPF4 Regulate Autophagic Turnover of the Golgi Apparatus,” EMBO Journal 43 (2024): 8, 10.1038/s44318-024-00131-3. PMC11250848 38822137

[72] J. Yang , Y. Dong , J. Xu , et al., “TM9SF3 Is a Golgi‐Resident ATG8‐Binding Protein Essential for Golgi‐Selective Autophagy,” Developmental Cell 60 (2025): 2862–2879.e8, 10.1016/j.devcel.2025.06.017. 40609542

[73] Y. Nibe‐Shirakihara , S. Honda , S. Arakawa , et al., “Optineurin Is an Adaptor Protein for Ubiquitinated Substrates in Golgi Membrane‐Associated Degradation,” Nature Communications 16 (2025): 8966, 10.1038/s41467-025-64400-3. PMC12537940 41115894

[74] H. Yamaguchi , S. Arakawa , T. Kanaseki , et al., “Golgi Membrane‐Associated Degradation Pathway in Yeast and Mammals,” EMBO Journal 35 (2016): 1991–2007, 10.15252/embj.201593191. PMC5282831 27511903

[75] S. Suklabaidya , S. Mohanty , I. E. Reider , et al., “Negative Feedback Regulation of STING Signaling by TAX1BP1‐Directed Golgiphagy,” Nature Communications 17 (2026): 2762, 10.1038/s41467-026-69422-z. PMC13018473 41673009

[76] Z. Zhang and C. Zhang , “Regulation of cGAS–STING Signalling and Its Diversity of Cellular Outcomes,” Nature Reviews Immunology 25 (2025): 425–444, 10.1038/s41577-024-01112-7. 39774812

[77] Z. Liu , G. Qin , J. Yang , et al., “Targeting Mitochondrial Degradation by Chimeric Autophagy‐Tethering Compounds,” Chemical Science 14 (2023): 11192–11202, 10.1039/d3sc03600f. PMC10583747 37860639

[78] F. Loos , W. Xie , V. Sica , et al., “Artificial Tethering of LC3 or p62 to Organelles Is Not Sufficient to Trigger Autophagy,” Cell Death & Disease 10 (2019): 771, 10.1038/s41419-019-2011-5. PMC6787181 31601788

[79] J. Kang , C. M. Li , N. Kim , J. Baek , and Y.‐K. Jung , “Non‐Autophagic Golgi‐LC3 Lipidation Facilitates TFE3 Stress Response Against Golgi Dysfunction,” EMBO Journal 43 (2024): 5085–5113, 10.1038/s44318-024-00233-y. PMC11535212 39284911

[80] T. M. Nthiga , B. Kumar Shrestha , E. Sjøttem , et al., “CALCOCO1 Acts With VAMP‐Associated Proteins to Mediate ER‐Phagy,” EMBO Journal 39 (2020): EMBJ2019103649, 10.15252/embj.2019103649. PMC7396842 32525583

[81] T. M. Nthiga , B. K. Shrestha , J.‐A. Bruun , K. B. Larsen , T. Lamark , and T. Johansen , “Regulation of Golgi Turnover by CALCOCO1‐Mediated Selective Autophagy,” Journal of Cell Biology 220 (2021), 10.1083/jcb.202006128. PMC8059076 33871553

[82] L. Lu , M. Tang , Z. Qi , et al., “Regulation of the Golgi Apparatus via GOLPH3‐Mediated New Selective Autophagy,” Life Sciences 253 (2020): 117700, 10.1016/j.lfs.2020.117700. 32335164

[83] A. Rahman , P. Lőrincz , R. Gohel , et al., “GMAP Is an Atg8a‐Interacting Protein that Regulates Golgi Turnover in Drosophila,” Cell Reports 39 (2022): 110903, 10.1016/j.celrep.2022.110903. PMC9637997 35649355

[84] R. Benyair , A. Eisenberg‐Lerner , and Y. Merbl , “Maintaining Golgi Homeostasis: A Balancing Act of Two Proteolytic Pathways,” Cells 11 (2022): 780, 10.3390/cells11050780. PMC8909885 35269404

[85] S. Schwabl and D. Teis , “Protein Quality Control at the Golgi,” Current Opinion in Cell Biology 75 (2022): 102074, 10.1016/j.ceb.2022.02.008. 35364487

[86] A. Eisenberg‐Lerner , R. Benyair , N. Hizkiahou , et al., “Golgi Organization Is Regulated by Proteasomal Degradation,” Nature Communications 11, no. 1 (2020): 409, 10.1038/s41467-019-14038-9. PMC6972958 31964869

[87] J. Diamantino , S. Murteira , S. Lawo , F. Kaschani , M. Kaiser , and D. Hellerschmied , “Selective Proteasomal Degradation From the Golgi Apparatus Membrane,“ bioRxiv: The Preprint Server for Biology (2026), 10.64898/2026.02.04.703791.

[88] O. Schmidt , Y. Weyer , V. Baumann , et al., “Endosome and Golgi‐Associated Degradation (EGAD) of Membrane Proteins Regulates Sphingolipid Metabolism,” EMBO Journal 38 (2019): e101433, 10.15252/embj.2018101433. PMC6669922 31368600

[89] Y. Weyer and D. Teis , “The Dsc Complex and Its Role in Golgi Quality Control,” Biochemical Society Transactions 52 (2024): 2023–2034, 10.1042/BST20230375. PMC11555709 39324639

[90] Y. Weyer , S. I. Schwabl , X. Tang , et al., “The Dsc Ubiquitin Ligase Complex Identifies Transmembrane Degrons to Degrade Orphaned Proteins at the Golgi,” Nature Communications 15 (2024): 9257, 10.1038/s41467-024-53676-6. PMC11513148 39461958

[91] S. M. Migliano and D. Teis , “ESCRT and Membrane Protein Ubiquitination,” in Progress in Molecular and Subcellular Biology, vol. 57 (Springer, 2018), 107–135, 10.1007/978-3-319-96704-2_4. 30097773

[92] A. Y. Minard , S. Winistorfer , L. Yu , and R. C. Piper , “K48‐UbiquitIn‐Dependent Proteases Cut‐up Post‐ER Proteins,” Nature Communications 17 (2026): 1669, 10.1038/s41467-026-68367-7. PMC12909987 41530178

[93] L. Buzuk and D. Hellerschmied , “Ubiquitin‐Mediated Degradation at the Golgi Apparatus,” Frontiers in Molecular Biosciences 10 (2023): 1197921, 10.3389/fmolb.2023.1197921. PMC10357820 37484530

[94] S. A. Pol , S. Liljenberg , J. Barr , et al., “Induced Degradation of SNAP‐Fusion Proteins,” RSC Chemical Biology 5 (2024): 1232–1247, 10.1039/d4cb00184b. PMC11494418 39444693

[95] Y. Hatoyama , Y. Homma , S. Hiragi , and M. Fukuda , “Establishment and Analysis of Conditional Rab1‐ and Rab5‐Knockout Cells Using the Auxin‐Inducible Degron System,” Journal of Cell Science 134 (2021): jcs259184, 10.1242/jcs.259184. 34817057

[96] F. T. Sumya , I. D. Pokrovskaya , Z. D’Souza , and V. V. Lupashin , “Acute COG Complex Inactivation Unveiled Its Immediate Impact on Golgi and Illuminated the Nature of Intra‐Golgi Recycling Vesicles,” Traffic 24 (2023): 52–75, 10.1111/tra.12876. PMC9969905 36468177

[97] Y. Zhang and J. Seemann , “Rapid Degradation of GRASP55 and GRASP65 Reveals Their Immediate Impact on the Golgi Structure,” Journal of Cell Biology 220 (2021): e202007052, 10.1083/jcb.202007052. PMC7735681 33301566

[98] K. Nishimura , T. Fukagawa , H. Takisawa , T. Kakimoto , and M. Kanemaki , “An Auxin‐Based Degron System for the Rapid Depletion of Proteins in Nonplant Cells,” Nature Methods 6 (2009): 917–922, 10.1038/nmeth.1401. 19915560

[99] A. Yesbolatova , Y. Saito , N. Kitamoto , et al., “The Auxin‐Inducible Degron 2 technology Provides Sharp Degradation Control in Yeast, Mammalian Cells, and Mice,” Nature Communications 11 (2020): 5701, 10.1038/s41467-020-19532-z. PMC7659001 33177522

[100] E. Ahat , J. Li , and Y. Wang , “New Insights Into the Golgi Stacking Proteins,” Frontiers in Cell and Developmental Biology 7 (2019): 131, 10.3389/fcell.2019.00131. PMC6660245 31380369

[101] C. Yeh , W. Huang , P. Hsu , et al., “The C‐Degron Pathway Eliminates Mislocalized Proteins and Products of Deubiquitinating Enzymes, EMBJ2020105846,” EMBO Journal 40 (2021): EMBJ2020105846, 10.15252/embj.2020105846. PMC8013793 33469951

[102] S. J. Tomlinson , S. L. Johnson , A. H. Kroskrity , et al., “Induced Ubiquitination Bypasses Canonical ERAD to Drive ER Protein Degradation,“ bioRxiv : The Preprint Server for Biology (2025), 10.1101/2025.11.28.691080.

[103] A. H. Erickson , “PA‐TM‐RING Proteins: A New Family of Endosomal Membrane Proteins,” FEBS Journal 278 (2011): 46–46, 10.1111/j.1742-4658.2010.07921.x. 21204313

[104] J. Poirson , H. Cho , A. Dhillon , et al., “Proteome‐Scale Discovery of Protein Degradation and Stabilization Effectors,” Nature 628 (2024): 878–886, 10.1038/s41586-024-07224-3. 38509365

[105] A. D. Cotton , D. P. Nguyen , J. A. Gramespacher , I. B. Seiple , and J. A. Wells , “Development of Antibody‐Based PROTACs for the Degradation of the Cell‐Surface Immune Checkpoint Protein PD‐L1,” Journal of the American Chemical Society 143 (2021): 593–598, 10.1021/jacs.0c10008. PMC8154509 33395526

[106] J. C. Christianson , E. Jarosch , and T. Sommer , “Mechanisms of Substrate Processing During ER‐Associated Protein Degradation,” Nature Reviews Molecular Cell Biology 24 (2023): 777–796, 10.1038/s41580-023-00633-8. 37528230

[107] J. A. Nathan , H. Tae Kim , L. Ting , S. P. Gygi , and A. L. Goldberg , “Why Do Cellular Proteins Linked to K63‐Polyubiquitin Chains Not Associate With Proteasomes?,” EMBO Journal 32 (2013): 552–565, 10.1038/emboj.2012.354. PMC3579138 23314748

[108] P.‐H. Kuhn , M. Voss , M. Haug‐Kröper , et al., “Secretome Analysis Identifies Novel Signal Peptide Peptidase‐Like 3 (SPPL3) Substrates and Reveals a Role of SPPL3 in Multiple Golgi Glycosylation Pathways*,” Molecular & Cellular Proteomics 14 (2015): 1584–1598, 10.1074/mcp.M115.048298. PMC4458722 25827571

[109] M. Voss , U. Künzel , F. Higel , et al., “Shedding of Glycan‐Modifying Enzymes by Signal Peptide Peptidase‐Like 3 (SPPL3) Regulates Cellular N‐Glycosylation,” EMBO Journal 33 (2014): 2890–2905, 10.15252/embj.201488375. PMC4282638 25354954

[110] J. Yang and H. Zhu , “Small‐Molecule Ligands Targeting Lysosome‐Shuttling Receptors and the Emerging Landscape of Lysosome‐Targeting Chimeras,” Journal of Medicinal Chemistry 68 (2025): 18040–18063, 10.1021/acs.jmedchem.5c01019. 40888077

[111] Z. Zhou , Q. Niu , X. Yu , C. Yang , and Z. Zhu , “Lysosome‐Targeting Chimeras (LYTACs): From Modular Design Principles to Diverse Therapeutic Applications,” Chemistry—A European Journal 32 (2026): e71255, 10.1002/chem.71255. 42298978

[112] B. Li , Y. Li , J. Zhang , et al., “Lysosome‐Targeting Chimeras Enable Targeted Protein Degradation,” Cell Chemical Biology 33 (2026): 433–460, 10.1016/j.chembiol.2026.01.008. 41713411

[113] B. Huang , M. Abedi , G. Ahn , et al., “Designed Endocytosis‐Inducing Proteins Degrade Targets and Amplify Signals,” Nature 638 (2025): 796‍–‍804, 10.1038/s41586-024-07948-2. PMC11839401 39322662

[114] C. Gustafsen , J. Vilstrup , M. Kristensen , et al., “Reshaping the Progranulin/Sortilin Interaction for Targeted Degradation of Extracellular Proteins,” Cell Chemical Biology 33 (2026): 490–505.e40, 10.1016/j.chembiol.2026.03.002. 41903545

[115] D. Zhang , J. Duque‐Jimenez , F. Facchinetti , et al., “Transferrin Receptor Targeting Chimeras for Membrane Protein Degradation,” Nature 638 (2025): 787–795, 10.1038/s41586-024-07947-3 PMC11839386 39322661

[116] Y. Shi , X. Liang , X. Xu , L. Li , and G. Chen , “Multitargeted Degradation of Cell Surface Receptors by Modular Glyco‐Nanosheets,” ACS Macro Letters 15 (2026): 991–998, 10.1021/acsmacrolett.6c00229. 42396733

[117] J. Luo , Q. Gao , K. Tan , et al., “Lysosome Targeting Chimaeras for Glut1‐Facilitated Targeted Protein Degradation,” Journal of the American Chemical Society 146 (2024): 17728–17737, 10.1021/jacs.4c02463. 38899504

[118] D. Xiao , J. Dong , F. Xie , et al., “Polyvalent Folate Receptor‐Targeting Chimeras for Degradation of Membrane Proteins,” Nature Chemical Biology 21 (2025): 1731–1741, 10.1038/s41589-025-01924-1. 40514550

[119] K. Wang , K. Wang , C. Wang , et al., “Development of Dual‐Receptor Lysosome‐Targeting Chimeras for Protein Degradation,” Journal of the American Chemical Society 147 (2025): 40751–40762, 10.1021/jacs.5c13695. 41127901

[120] L. Zhu , H. Liu , X. Zeng , et al., “Development of Glucagon‐Like Peptide‐1 Lysosomal Targeting Chimeras for Degradation of Extracellular and Membrane Proteins,” European Journal of Medicinal Chemistry 303 (2026): 118473, 10.1016/j.ejmech.2025.118473. 41401593

[121] K. Pance , J. A. Gramespacher , J. R. Byrnes , et al., “Modular Cytokine Receptor‐Targeting Chimeras for Targeted Degradation of Cell Surface and Extracellular Proteins,” Nature Biotechnology 41 (2023): 273–281, 10.1038/s41587-022-01456-2. PMC9931583 36138170

[122] Q. Wang , X. Yang , R. Yuan , et al., “A Co‐Assembly Platform Engaging Macrophage Scavenger Receptor a for Lysosome‐Targeting Protein Degradation,” Nature Communications 15 (2024): 1663, 10.1038/s41467-024-46130-0. PMC10891067 38396109

[123] W. Sun , H. Zhang , W. Xie , et al., “Development of Integrin‐Facilitated Bispecific Aptamer Chimeras for Membrane Protein Degradation,” Journal of the American Chemical Society 146 (2024): 25490–25500, 10.1021/jacs.4c04794. 39226482

[124] S. R. Pfeffer , “Multiple Routes of Protein Transport From Endosomes to the Trans Golgi Network,” FEBS Letters 583 (2009): 3811–3816, 10.1016/j.febslet.2009.10.075. PMC2787657 19879268

[125] M. F. Coutinho , M. J. Prata , and S. Alves , “Mannose‐6‐Phosphate Pathway: A Review on Its Role in Lysosomal Function and Dysfunction,” Molecular Genetics and Metabolism 105 (2012): 542–550, 10.1016/j.ymgme.2011.12.012. 22266136

[126] B. Doray , P. Ghosh , J. Griffith , H. J. Geuze , and S. Kornfeld , “Cooperation of GGAs and AP‐1 in Packaging MPRs at the Trans‐Golgi Network,” Science 297 (2002): 1700–1703, 10.1126/science.1075327. 12215646

[127] J. Hirst , M. R. Lindsay , and M. S. Robinson , “Golgi‐Localized, γ‐Ear‐Containing, ADP‐Ribosylation Factor‐Binding Proteins: Roles of the Different Domains and Comparison With AP‐1 and Clathrin,” Molecular Biology of the Cell 12 (2001): 3573–3588, 10.1091/mbc.12.11.3573. PMC60277 11694590

[128] P. Ghosh , N. M. Dahms , and S. Kornfeld , “Mannose 6‐Phosphate Receptors: New Twists in the Tale,” Nature Reviews Molecular Cell Biology 4 (2003): 202–213, 10.1038/nrm1050. 12612639

[129] C. N. Arighi , L. M. Hartnell , R. C. Aguilar , C. R. Haft , and J. S. Bonifacino , “Role of the Mammalian Retromer in Sorting of the Cation‐Independent Mannose 6‐Phosphate Receptor,” Journal of Cell Biology 165 (2004): 123–133, 10.1083/jcb.200312055. PMC2172094 15078903

[130] R. Pohlmann , G. Nagel , A. Hille , et al., “Mannose 6‐Phosphate Specific Receptors: Structure and Function,” Biochemical Society Transactions 17 (1989): 15–16, 10.1042/bst0170015. 2541033

[131] J.‐J. P. Kim , L. J. Olson , and N. M. Dahms , “Carbohydrate Recognition by the Mannose‐6‐Phosphate Receptors,” Current Opinion in Structural Biology 19 (2009): 534–542, 10.1016/j.sbi.2009.09.002. PMC2771201 19801188

[132] M. K. Hancock , D. J. Haskins , G. Sun , and N. M. Dahms , “Identification of Residues Essential for Carbohydrate Recognition by the Insulin‐Like Growth Factor II/Mannose 6‐Phosphate Receptor,” Journal of Biological Chemistry 277 (2002): 11255–11264, 10.1074/jbc.M109855200. 11799115

[133] R. N. Bohnsack , X. Song , L. J. Olson , et al., “Cation‐Independent Mannose 6‐Phosphate Receptor,” Journal of Biological Chemistry 284 (2009): 35215–35226, 10.1074/jbc.M109.056184. PMC2787381 19840944

[134] L. J. Olson , A. C. Castonguay , Y. Lasanajak , et al., “Identification of a Fourth Mannose 6‐Phosphate Binding Site in the Cation‐Independent Mannose 6‐Phosphate Receptor,” Glycobiology 25 (2015): 591–606, 10.1093/glycob/cwv001. PMC4410830 25573276

[135] S. J. Stachel , A. T. Ginnetti , S. A. Johnson , et al., “Identification of Potent Inhibitors of the Sortilin‐Progranulin Interaction,” Bioorganic & Medicinal Chemistry Letters 30 (2020): 127403, 10.1016/j.bmcl.2020.127403. 32738972

[136] L. J. Olson , S. K. Misra , M. Ishihara , et al., “Allosteric Regulation of Lysosomal Enzyme Recognition by the Cation‐Independent Mannose 6‐Phosphate Receptor,” Communications Biology 3, no. 1 (2020): 498, 10.1038/s42003-020-01211-w. PMC7481795 32908216

[137] N. R. M. Reintjens , E. Tondini , C. Vis , et al., “Multivalent, Stabilized Mannose‐6‐Phosphates for the Targeted Delivery of Toll‐Like Receptor Ligands and Peptide Antigens,” ChemBioChem 22 (2021): 434–440, 10.1002/cbic.202000538. PMC7894537 32864819

[138] C. M. Stevens , Y. Zhou , P. Teng , L. N. Rault , Y. Liao , and W. Tang , “Development of Oligomeric Mannose‐6‐Phosphonate Conjugates for Targeted Protein Degradation,” ACS Medicinal Chemistry Letters 14 (2023): 719–726, 10.1021/acsmedchemlett.2c00479. PMC10258825 37312839

[139] B. Zhang , R. K. Brahma , L. Zhu , et al., “Insulin‐Like Growth Factor 2 (IGF2)‐Fused Lysosomal Targeting Chimeras for Degradation of Extracellular and Membrane Proteins,” Journal of the American Chemical Society 145 (2023): 24272–24283, 10.1021/jacs.3c08886. 37899626

[140] J. L. Yang , S. A. Yamada‐Hunter , L. Labanieh , et al., “Directed Evolution of Genetically Encoded LYTACs for Cell‐Mediated Delivery,” Proceedings of the National Academy of Sciences 121 (2024): e2320053121, 10.1073/pnas.2320053121. PMC10990137 38513100

[141] M. S. Nielsen , P. Madsen , E. I. Christensen , et al., “The Sortilin Cytoplasmic Tail Conveys Golgi–Endosome Transport and Binds the VHS Domain of the GGA2 Sorting Protein,” EMBO Journal 20 (2001): 2180–2190, 10.1093/emboj/20.9.2180. PMC125444 11331584

[142] K. A. Mitok , M. P. Keller , and A. D. Attie , “Sorting Through the Extensive and Confusing Roles of Sortilin in Metabolic Disease,” Journal of Lipid Research 63 (2022): 100243, 10.1016/j.jlr.2022.100243. PMC9356209 35724703

[143] M. Canuel , Y. Libin , and C. R. Morales , “The Interactomics of Sortilin: An Ancient Lysosomal Receptor Evolving New Functions,” Histology and Histopathology 24 (2009): 481–492, 10.14670/HH-24.481. 19224451

[144] E. M. Quistgaard , P. Madsen , M. K. Grøftehauge , P. Nissen , C. M. Petersen , and S. S. Thirup , “Ligands Bind to Sortilin in the Tunnel of a Ten‐Bladed β‐Propeller Domain,” Nature Structural & Molecular Biology 16 (2009): 96–98, 10.1038/nsmb.1543. 19122660

[145] S. Itoh , K. Mizuno , M. Aikawa , and E. Aikawa , “Dimerization of Sortilin Regulates Its Trafficking to Extracellular Vesicles,” Journal of Biological Chemistry 293 (2018): 4532–4544, 10.1074/jbc.RA117.000732. PMC5868269 29382723

[146] N. Leloup , P. Lössl , D. H. Meijer , et al., “Low pH‐Induced Conformational Change and Dimerization of Sortilin Triggers Endocytosed Ligand Release,” Nature Communications 8, no. 1 (2017): 1708, 10.1038/s41467-017-01485-5. PMC5700061 29167428

[147] D. Januliene , J. L. Andersen , J. A. Nielsen , et al., “Acidic Environment Induces Dimerization and Ligand Binding Site Collapse in the Vps10p Domain of Sortilin,” Structure 25 (2017): 1809–1819.e3, 10.1016/j.str.2017.09.015. 29107483

[148] E. Trabjerg , N. Abu‐Asad , Z. Wan , F. Kartberg , S. Christensen , and K. D. Rand , “Investigating the Conformational Response of the Sortilin Receptor Upon Binding Endogenous Peptide‐ and Protein Ligands by HDX‐MS,” Structure 27 (2019): 1103–1113.e3, 10.1016/j.str.2019.04.006. 31104815

[149] B. M. Tyler‐McMahon , M. Boules , and E. Richelson , “Neurotensin: Peptide for the Next Millennium,” Regulatory Peptides 93 (2000): 125–136, 10.1016/s0167-0115(00)00183-x. 11033059

[150] J. L. Andersen , T. J. Schrøder , S. Christensen , et al., “Identification of the First Small‐Molecule Ligand of the Neuronal Receptor Sortilin and Structure Determination of the Receptor‐Ligand Complex,” Acta Crystallographica. Section D, Biological Crystallography 70 (2014): 451–460, 10.1107/S1399004713030149. PMC3940197 24531479

[151] T. J. Schrøder , S. Christensen , S. Lindberg , et al., “The Identification of AF38469: An Orally Bioavailable Inhibitor of the VPS10P Family Sorting Receptor Sortilin,” Bioorganic & Medicinal Chemistry Letters 24 (2014): 177–180, 10.1016/j.bmcl.2013.11.046. 24355129

[152] D. Reczek , M. Schwake , J. Schröder , et al., “LIMP‐2 Is a Receptor for Lysosomal Mannose‐6‐Phosphate‐Independent Targeting of β‐Glucocerebrosidase,” Cell 131 (2007): 770–783, 10.1016/j.cell.2007.10.018. 18022370

[153] “Programs ‐ Lycia Therapeutics,” Can Be Found Under, accessed October 12, 2026, https://lyciatx.com/pipeline/programs/.

[154] G. Ahn , N. M. Riley , R. A. Kamber , et al., “Elucidating the Cellular Determinants of Targeted Membrane Protein Degradation by Lysosome‐Targeting Chimeras,” Science 382 (2023): 2023, 10.1126/science.adf6249. PMC10766146 37856615

[155] J. M. Mata , E. van der Nol , and S. J. Pomplun , “Advances in Ultrahigh Throughput Hit Discovery With Tandem Mass Spectrometry Encoded Libraries,” Journal of the American Chemical Society 145 (2023): 19129–19139, 10.1021/jacs.3c04899. PMC10472510 37556835

[156] A. L. Satz , A. Brunschweiger , M. E. Flanagan , et al., “DNA‐Encoded Chemical Libraries,” Nature Reviews Methods Primers 2 (2022): 3, 10.1038/s43586-021-00084-5.

[157] C. Gauthier , K. El Cheikh , I. Basile , et al., “Cation‐Independent Mannose 6‐Phosphate Receptor: From Roles and Functions to Targeted Therapies,” Journal of Controlled Release 365 (2024): 759–772, 10.1016/j.jconrel.2023.12.014. 38086445

[158] P. J. Thul , L. Åkesson , M. Wiking , et al., “A Subcellular Map of the Human Proteome,” Science 356 (2017): eaal3321, 10.1126/science.aal3321. 28495876

[159] R. Fasimoye , W. Dong , R. S. Nirujogi , et al., “Golgi‐IP, a Tool for Multimodal Analysis of Golgi Molecular Content,” Proceedings of the National Academy of Sciences 120 (2023): e2219953120, 10.1073/pnas.2219953120. PMC10193996 37155866

[160] J. P. Schessner , V. Albrecht , A. K. Davies , P. Sinitcyn , and G. H. H. Borner , “Deep and Fast Label‐Free Dynamic Organellar Mapping,” Nature Communications 14 (2023): 5252, 10.1038/s41467-023-41000-7. PMC10465578 37644046

[161] M. Y. Hein , D. Peng , V. Todorova , et al., “Global Organelle Profiling Reveals Subcellular Localization and Remodeling at Proteome Scale,” Cell 188 (2025): 1137–1155.e20, 10.1016/j.cell.2024.11.028. 39742809

[162] A. Geladaki , N. Kočevar Britovšek , L. M. Breckels , et al., “Combining LOPIT With Differential Ultracentrifugation for High‐Resolution Spatial Proteomics,” Nature Communications 10 (2019): 331, 10.1038/s41467-018-08191-w. PMC6338729 30659192

[163] C. D. Go , J. D. R. Knight , A. Rajasekharan , et al., “A Proximity‐Dependent Biotinylation Map of a Human Cell,” Nature 595 (2021): 120–124, 10.1038/s41586-021-03592-2. 34079125

[164] C. A. Franziscus , A. Ferrand , O. Biehlmaier , A. Schmidt , and A. Spang , “SPEx: Compartment‐Resolved Proteomics via Expansion Microscopy–Guided Microdissection,” eLife revised preprint (2026), 10.7554/eLife.111393.1.

